# Prediction of prokinetic agents in critically ill patients with feeding intolerance: a prospective observational clinical study

**DOI:** 10.3389/fnut.2023.1244517

**Published:** 2023-10-27

**Authors:** Guangxuan Lv, Tao Zhang, Luping Wang, Xin Fu, Yucong Wang, Hua Yao, Huang Fang, Xiaoxiao Xia, Jing Yang, Bo Wang, Zhongwei Zhang, Xiaodong Jin, Yan Kang, Yisong Cheng, Qin Wu

**Affiliations:** Department of Critical Care Medicine, West China Hospital, Sichuan University, Chengdu, China

**Keywords:** gastric antrum echodensity, feeding intolerance, prokinetic agents, enteral nutrition, critical illness

## Abstract

**Background:**

Prokinetic agents are currently considered the first-line therapy to improve gastric emptying when feeding intolerance occurred in critically ill adults. In this study, we developed a technique to assess the feasibility of predicting prokinetic agent efficacy in critically ill patients.

**Methods:**

The first images of each patient were obtained after EFI had occurred but before the first dose of prokinetic agents was administered and additional images were obtained every morning until the seventh day. The gastric antrum echodensity was recorded based on grayscale values (50th percentile, ED50; 85th percentile, ED85; mean, ED_mean_) and daily energy and protein intake was collected as the judgment for effective and ineffective group. A receiver operating characteristic curve was analyzed to distinguish the thresholds between the two groups and thus determine the ability of the gastric antrum echodensity to predict the efficacy of prokinetic agents.

**Results:**

In total, 83 patients were analyzed. Patients in the ineffective group had a higher ED_50_ (58.13 ± 14.48 vs. 49.88 ± 13.78, *p* < 0.001, difference 95% CI: 5.68, 10.82), ED_85_ (74.81 ± 16.41 vs. 65.70 ± 16.05, *p* < 0.001, difference 95% CI:6.16, 12.05), and ED_mean_ (60.18 ± 14.31 vs. 51.76 ± 14.08, *p* < 0.001, difference 95% CI: 5.85, 11.00) than those in the effective group. Patients in the effective group more easily reached the target energy 16.21 ± 7.98 kcal/kg vs. 9.17 ± 6.43 kcal/kg (*p* < 0.001), 0.72 ± 0.38 g/kg vs. 0.42 ± 0.31 g/kg (*p* < 0.001) than in the ineffective group intake by day.

**Conclusion:**

The gastric antrum echodensity might serve as a tool for judging the efficacy of prokinetic agents, helping clinicians to decide whether to use prokinetic agents or place a post-pyloric tube when feeding intolerance occurs in critically ill patients.

**Clinical trial registration:**http://www.chictr.org.cn/addproject2.aspx, ChiCTR2200058373. Registered 7 April 2022.

## 1. Introduction

Gastrointestinal dysfunction is common in critically ill patients (1). There are several risk factors associated with gastrointestinal dysfunction. A comprehensive analysis of a multicenter and multiyear database revealed that various diseases can lead to gastrointestinal dysfunction and feeding intolerance. Among these diseases, burns and gastrointestinal disorders pose the highest risk for enteral feeding intolerance (2, 3). In the ICU, sedation and analgesia are commonly used treatment measures, but these medications can have a direct or indirect impact on gastric emptying (4–6). Additionally, excessive fluid resuscitation and gastrointestinal tissue edema can contribute to reduced gastrointestinal contractility and motility (7). Moreover, brain-gut axis dysfunction also play a import role in the pathogenesis of Gastrointestinal dysfunction (8). Other factors such as hypoxia, acidosis, and disturbances in the internal environment can also influence gastrointestinal function. Consequently, these factors can result in feeding intolerance when enteral nutrition is administered, either directly or indirectly.

Early enteral nutrition is reportedly one of the main measures used to restore the gastrointestinal barrier and function (9, 10). One of the general complications encountered during the administration of enteral nutrition is enteral feeding intolerance (EFI), which occurs in more than 30% of critically ill patients and is often accompanied by worse outcomes compared with patients who tolerate enteral feeding (11, 12). Prokinetic agents are currently considered the first-line therapy for EFI, especially for patients with delayed gastric emptying and a high gastric residual volume (GRV) given the issues relating to parenteral nutrition and post-pyloric feeding in these patients (9). In spite of the side effects of the prokinetic agents such as erythromycin, metoclopramide, domperidone, cisapride, and itopride, we must still rely on these drugs to improve feeding performance in patients who are at high risk for aspiration and with critical illness-associated gastric motility dysfunction.

In addition to QT prolongation, one of the main issues associated with prokinetics is that these drugs become less effective during prolonged administration (13, 14). Moreover, not all patients respond to prokinetic therapy because of individual variations or other factors. Nguyen et al. (15) found that the effectiveness of prokinetic agents in ensuring successful feeding during EFI progressively declined over 7 days. These patients must be identified as early as possible to reduce unnecessary drug use and avert side effects. However, standard techniques with which to determine whether a prokinetic agent will be effective have not been established. Some studies have used the presence of high gastric residual volume (GRV) or other symptoms of feeding intolerance as the criterion for efficacy; however, high GRV has been an inconsistent index between different studies, and proving whether it can be used to accurately determine feeding intolerance requires further scientific evidence (15, 16).

Some recent studies have used ultrasonography for daily evaluation of gastrointestinal function, implementing a gastrointestinal and urinary tract sonography protocol to assess acute gastrointestinal injury (AGI) of critically ill patients (17, 18). In our previous study, we developed a new ultrasonography technique involving measurement of the gastric antrum echodensity to explore the relationship between the grade of AGI and assess its ability to judge feeding intolerance. Our data showed that the gastric antrum echodensity was highly correlated to the severity of AGI and could serve as a novel tool to predict feeding intolerance (19).

In the present study, we explored the relationship between the gastric antrum echodensity and the efficacy of prokinetic agents in critically ill patients to determine whether the gastric antrum echodensity can be used as a novel tool for choosing whether to administer prokinetic agents or place a nasointestinal tube for enteral feeding. We expected that the gastric antrum echodensity would be higher in patients for whom prokinetic agents are ineffective and that a higher echodensity may serve as a valid index for predicting prokinetic agent efficacy.

## 2. Methods

### 2.1. Ethical approval

This prospective study was conducted in the intensive care unit (ICU) from April to August 2022.

Ethical approval was obtained from the local Institutional Review Board (Ethics Approval Committee number: 2022S424) and registered on the China Clinical Trial Registry (No. ChiCTR2200058373) and informed consent was obtained from each patient or their next-of-kin.

### 2.2. Study population

Patients undergoing mechanical ventilation were eligible if they had begun enteral feeding by a gastric tube more than 48 h previously, had developed feeding intolerance, and had received at least one prokinetic agent in the ICU. Patients were excluded if they had not begun enteral nutrition, had not developed feeding intolerance, had received enteral nutrition orally or by post-pyloric feeding at the beginning of treatment, did not have clear point-of-care ultrasonography (POCUS) images of the gastric antrum, or were expected to die within 48 h after admission to the ICU. Patients aged <18 years, pregnant patients, and patients who refused to participate in the study were also excluded.

Enteral feeding intolerance was defined as the presence of persistent vomiting/regurgitation or a high GRV resulting in forced interruption of enteral feeding. If any visible reflux of gastric contents occurred, vomiting/regurgitation was diagnosed. The GRV was considered high if it exceeded 200 mL in a single measurement (20, 21).

Prokinetic agents were considered effective if the energy to target energy ratio was ≥50% on day 7 of treatment (effective group). Prokinetic agents were deemed ineffective if the energy to target energy ratio was <50% on day 7 or if the patients transitioned to post-pyloric feeding after using the agents despite reaching the energy goal (ineffective group).

### 2.3. Nutrition protocols and prokinetic agents

Enteral feeding was initiated as soon as possible (within 24–48 h) after ICU admission if there was no contraindication for enteral nutrition. Enteral nutrition was begun at 20 mL/h, and the feeding rate was gradually increased if the patients tolerated the feeding well; the feeding rate did not exceed 150 mL/h. If deemed appropriate based on daily clinical assessment, the patients were transitioned to a volume-based feeding strategy using the enhanced protein-energy provision via the enteral route feeding protocol (PEP uP protocol) during the next and subsequent day (22–24).

The daily caloric prescription was determined according to the patient’s clinical condition and aimed to achieve 25 kcal/kg per 24-h period based on the estimated dry weight of the patient at ICU admission while the prescription was adjusted by adding 25% of the difference between the estimated dry weight and the ideal body weight for obese patients (BMI ≥ 30) by using a standard formula to calculate the ideal body weight (25). The target protein intake was 1.2–1.5 g/kg of body weight per day.

We chose a semi-elemental, concentrated feeding solution (Enteral Nutrition Emulsion, TPF-T) that would be useful in both full volume and trophic fed patients (Wuxi, Jiangsu province, China). The dietitian could suggest changes after the protocol was started based on further assessment and to initiate a protein supplement.

The usage of prokinetic agents were decided by clinicians. Patients with EFI received either metoclopramide (10 mg administered as a 50-mL IV infusion over 30 min every 8 h) or domperidone (10 mg administered via tube feeding every 8 h) or mosapride (10 mg administered via tube feeding every 8 h) or the combination of two or three agents. The dose of IV metoclopramide was adjusted by the level of creatinine clearance (creatinine clearance ≤40 mL/min with 50% of normal dose and clearance ≤10 mL/min or undergoing dialysis or continuous renal replacement techniques with 25% of normal dose in patients) (26).

### 2.4. Point-of-care ultrasonography examination and echodensity measurement

Point-of-care ultrasonography (POCUS) was used to measure the gastric antrum echodensity in all critically ill patients in this study. A physician with 3 years of experience in ultrasonography performed the scans before and after the administration of prokinetic agents. POCUS was performed using a curvilinear probe with specific parameters set according to a previous study (19).

The following was the procedure of examination and echodensity measurement in the study.

- The time of point-of-care ultrasonography: The first images of each patient were obtained after EFI had occurred but before the first dose of prokinetic agents was administered. Additional images were obtained every morning until either the seventh day or the patients were discharged or transferred from the ICU.
- The type and parameters of ultrasonic device: A 1- to 5-MHz curvilinear probe (CX50; Philips, Bothell, WA, United States) was used to visualize the gastric antrum. The POCUS parameters were preset in accordance with our previous study (transverse gain compensation set to zero, time gain compensation adjusted to the maximum, ultrasonic gain set to 16 accompanied by a frequency of 38 Hz and depth of 13 cm) (19). B-mode images were taken at the end of gastric antrum contraction for echodensity measurement.
- The standard position of patients during the measurement: The patients were placed in the supine position. In the epigastric area, the left lobe of the liver, superior mesenteric vein, and abdominal aorta were used as the markers to image the gastric antrum.
- Screen images for further analysis: The ultrasound images that clearly showed the structure of the gastric antrum were screened for further analysis.
- The obtaining of grayscales of ED_50_, ED_85_, and ED_mean_: The largest artifact-free area was selected between the mucosal and serosal layers of the gastric antrum (but not including these layers) for histogram analysis. Image processing was performed in accordance with our previously published study [the color pattern was converted to 8-bit, and the largest artifact-free area was selected using ImageJ software 1.41o (National Institutes of Health, Bethesda, MD, United States)]. Histogram analysis was used to generate the grayscale frequency distribution of the echodensity of the selected region by the software. According to the histogram analysis, we defined ED_50_ as the 50th percentile, ED_85_ as the 85th percentile, and ED_mean_ as the mean value of the grayscale distribution. Two observers who were blinded to the patient grouping measured the gastric antrum echodensity to avoid subjective bias.

### 2.5. Data collection

The following baseline characteristics of all critically ill patients were collected within the first 24 h after admission to the ICU: age, sex, height, weight, body mass index (BMI), reason for ICU admission, Acute Physiology and Chronic Health Evaluation II (APACHE II) score, and Sequential Organ Failure Assessment (SOFA) score. Laboratory examinations were also conducted; these included white blood cell and platelet counts; measurements of the concentrations of biochemical indexes (total bilirubin, hemoglobin, serum creatinine, glucose, and albumin), procalcitonin, C-reactive protein, interleukin-6, and lactic acid; and measurement of the activated partial thromboplastin time, prothrombin time, and D-dimer. All patients’ gastrointestinal function was estimated daily; this included determination of the AGI grade (I–IV), Gastrointestinal Failure (GIF) score, and Gastrointestinal Dysfunction Score (GIDS) (19, 20). Data regarding the daily energy and protein intake from the first to seventh days of using prokinetic agents and placing the nasointestinal tube were also collected. Other data collected included the use of dexmedetomidine, propofol, midazolam, fentanyl, sufentanil, remifentanil, or norepinephrine for sedation/analgesia; use of other vasoactive agents; 28-day mortality rate; hospital mortality rate; length of ICU stay; and length of hospital stay.

### 2.6. Statistical analysis

A sample size of 88 patients was estimated to provide 90% power at a 2-sided alpha of 5% to demonstrate a mean difference of 10 with a standard deviation for both groups of 13 based on our pilot study which demonstrated an 90% relative reduction in the mean difference of 10 (62 vs. 52) after accounting for 20% dropout (PASS v21.0.3, NCSS software, Kaysville, United States).

Continuous variables are expressed as mean ± standard deviation or median (interquartile range) and were compared using Student’s t-test or the Mann–Whitney U test. Categorical variables are expressed as frequency (percentage) and were compared with the chi-square test or Fisher’s exact test as appropriate. Receiver operator characteristic (ROC) curve analysis and the Youden index were used to determine the ability of the gastric antrum echodensity to discriminate the efficacy of prokinetic agents. The baseline characteristics were compared between high-risk and low-risk patients according to the optimal gastric antrum echodensity cutoff value of the ED_50_ on the first day to distinguish the efficacy of the agents. The relationship between the efficacy of prokinetic agents and the patients’ clinical characteristics (including the gastric antrum echodensity) was assessed by logistic regression. Only variables with statistical significance (*p* < 0.05) were included in the multivariable models to identify those variables associated with the efficacy of prokinetic agents. To reach the final parsimonious model during the process of multivariable modeling, we performed forward elimination of variables that were not statistically significant. A two-tailed *p-*value of < 0.05 was deemed statistically significant. Subgroup analysis of the type of prokinetic agents was performed between the effective group and ineffective group. All statistical analyses were performed using Origin 2021 (OriginLab, Northampton, MA, United States), SPSS 26.0 (IBM Corp., Armonk, NY, United States), and MedCalc 20.1.0 (MedCalc Software, Ostend, Belgium).

## 3. Results

### 3.1. Patient demographics and disposition

**Figure 1:**
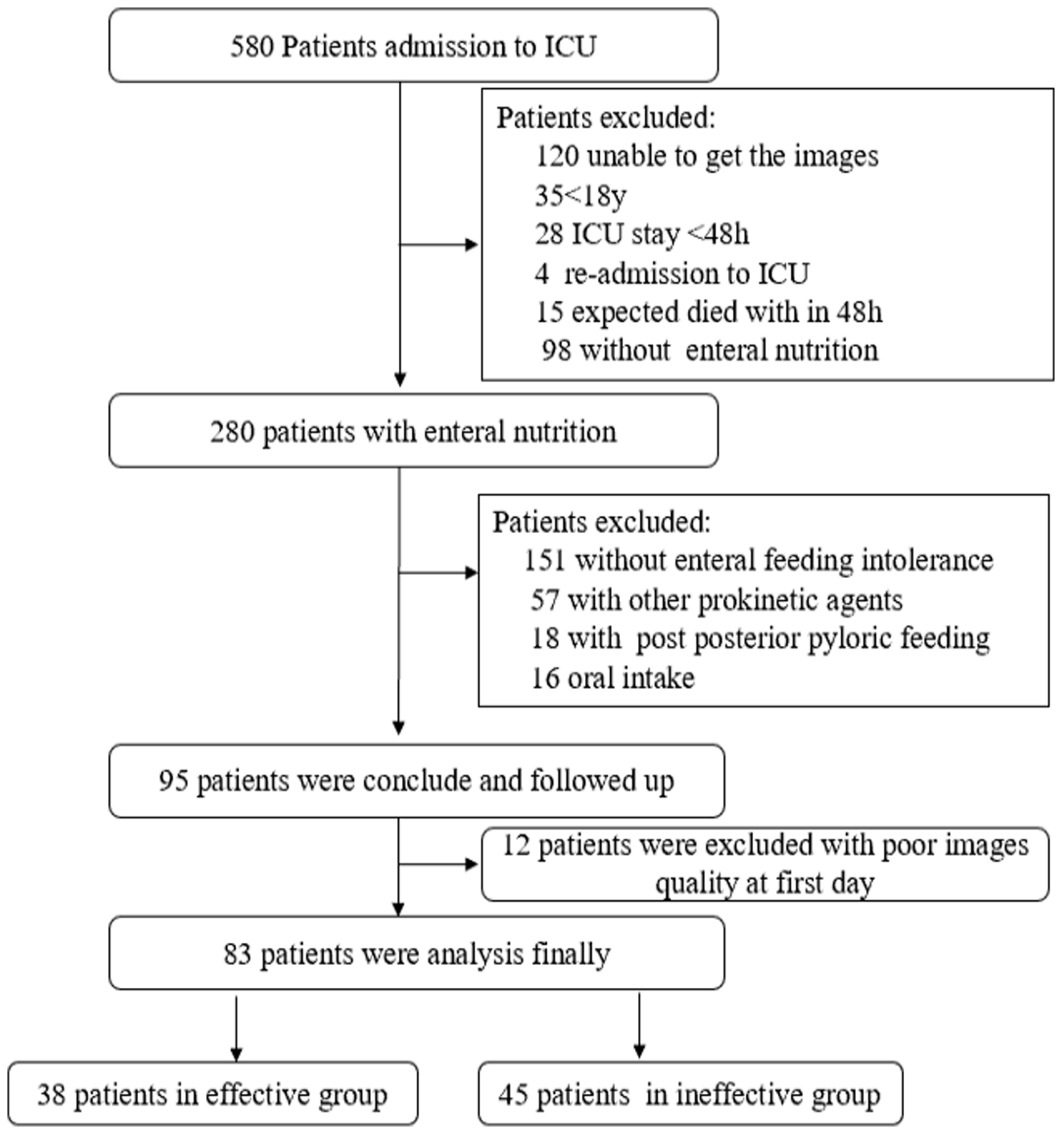
Flowchart and enrollment of patients.

From April to August 2022, 580 patients from the ICU were screened. Among them, 485 patients did not meet the inclusion criteria: Point-of-care ultrasonography imaging of the gastric antrum could not be performed because of abdominal surgery or abdominal bloating (*n* = 120), the patients were < 18 years old (*n* = 35), the patients stayed in the ICU for <48 h (*n* = 28), the patients were expected to die within 48 h after ICU admission (*n* = 15), the patients were readmitted to the ICU (*n* = 4), enteral nutrition was not initiated (*n* = 98), the patients did not develop feeding intolerance (*n* = 151), oral intake was initiated (*n* = 16), and enteral feeding was initiated by post-pyloric feeding (*n* = 18). Therefore, 95 patients who began enteral nutrition feeding by a gastric tube, developed feeding intolerance, and received prokinetic agents were followed up. With poor quality of images during the first day, 12 patients were excluded and 83 patients were performed in the analyses finally (Figure 1).

The most common cause of ICU admission were respiratory and neurologic diseases (*n* = 16, 19.28%) and the followed by sepsis (*n* = 14, 16.87%) and trauma (*n* = 11, 13.25%) among the patients. There were 11 patients of cardiovascular diseases and 16 patients for other diseases which were included thrombotic thrombocytopenic purpura, thermoplegia, SLE (systemic lupus erythematosus), leukemia, acute hepatic failure, CKD (chronic kidney disease), infections of laryngeal pharynges, infections with uncertain reason.

Among the 83 patients, 58 (69.88%) were men. The mean overall age was 56.14 ± 16.11 year, and the mean BMI was 23.43 ± 4.26 kg/m^2^. The mean overall mean SOFA score and APACHE II score were 9.72 ± 3.71 and 17.29 ± 8.40, respectively. The 28-day mortality rate and overall in-hospital mortality rate was 20.48% (*n* = 17) and 30.12% (*n* = 25), respectively. Of the 83 patients, 45 patients’ energy to target energy ratio was <50% on the seventh day, while 38 patients’ energy to target energy ratio was ≥50%. Nineteen patients (42.22%) were transitioned to post-pyloric feeding.

According to the energy goal and the proportion of patients who were transitioned to post-pyloric feeding as predefined in the study protocol, the whole cohort was divided into an effective group (*n* = 38, 45.78%) and an ineffective group (*n* = 45, 54.22%) according to the efficacy of prokinetic agents. There was no significant difference between the effective group and the ineffective group in age (54.72 ± 18.30 vs. 57.41 ± 14.29 years, *p* = 0.451, difference 95% CI, −4.38, 9.76), BMI (23.12 ± 4.49 vs. 23.71 ± 4.08 kg/m^2^, *p* = 0.280, difference 95% CI, −1.29, 2.45), APACHE II score (17.03 ± 7.41 vs. 17.52 ± 9.28, *p* = 0.790, difference 95% CI, −3.20, 4.20), or SOFA score (9.54 ± 3.72 vs. 9.89 ± 3.72, *p* = 0.672, difference 95% CI, −1.28, 1.97). The reason for admission to the ICU and the laboratory indicators between the two groups were shown in Table 1. The baseline characteristics are shown in Table 1.

**Table 1:** Baseline and characteristics of patients in prokinetic agents effective and ineffective group.

	Total (*n* = 83)	Effective group (*n* = 38)	Infective group (*n* = 45)	*p* value	OR/difference 95% CI
Age (years), mean ± SD	56.14 ± 16.11	54.72 ± 18.03	57.41 ± 14.29	0.451	(−4.38, 9.76)
Male sex, no. (%)	58 (69.88)	25 (65.79)	33 (73.33)	0.280	1.68 (0.65, 4.32)
BMI, mean ± SD	23.43 ± 4.26	23.12 ± 4.49	23.71 ± 4.08	0.542	(−1.29, 2.45)
APHACHE II score, mean ± SD	17.29 ± 8.40	17.03 ± 7.41	17.52 ± 9.28	0.790	(−3.20, 4.20)
SOFA score, mean ± SD	9.72 ± 3.71	9.54 ± 3.73	9.89 ± 3.72	0.672	(−1.28, 1.97)
**Reasons for ICU admission, *n* (%)**					
Respiratory diseases^a^	16 (19.28)	8 (21.05)	8 (17.78)	0.788	1.16 (0.39, 3.46)
Neurologic diseases^b^	16 (19.28)	11 (28.95)	5 (11.11)	0.052	0.40 (0.15, 1.06)
Sepsis	14 (16.87)	2 (6.26)	12 (26.67)	0.007	5.32 (1.27, 22.30)
Cardiovascular diseases^c^	10 (12.05)	4 (10.53)	6 (13.33)	0.637	1.33 (0.41, 4.37)
Trauma	11 (13.25)	8 (21.05)	3 (6.67)	0.07	0.33 (0.10, 1.17)
Other^d^	16 (19.28)	5 (13.16)	11 (24.44)	0.190	1.77 (0.74, 4.27)
**Co-morbidities, no. (%)**					
Diabetes mellitus	25 (30.12)	8 (21.05)	17 (37.77)	0.070	1.88 (0.96, 3.88)
Hypertension	28 (33.73)	14 (36.84)	14 (31.11)	0.695	0.89 (0.49, 1.62)
**Laboratory tests at admission, mean ± SD**					
White blood cell (*10^9^/L)	11.86 ± 6.83	11.50 ± 8.01	12.17 ± 5.67	0.470	(−2.33, 3.67)
Hemoglobin (g/L)	95.42 ± 22.34	94.59 ± 20.54	96.16 ± 24.04	0.706	(−8.26, 11.40)
Platelet (*10^9^/L)	172.16 ± 106.51	172.51 ± 86.89	171.84 ± 122.44	0.981	(−47.56, 46.22)
Albumin (g/L)	32.34 ± 5.58	32.57 ± 6.53	32.14 ± 4.66	0.647	(−3.28, 1.27)
Total bilirubin (umol/L)	19.70 ± 28.19	18.16 ± 25.63	21.07 ± 30.51	0.726	(−2.88, 2.08)
Serum creatinine (umol/L)	132.92 ± 129.88	102.82 ± 73.13	159.59 ± 160.91	0.062	(0.98, 112.56)
Glucose (mmol/L)	11.00 ± 5.85	10.18 ± 5.82	11.73 ± 5.85	0.162	(−1.01, 4.10)
C-reactive protein (mg/L)	115.60 ± 100.912	100.16 ± 80.96	129.61 ± 115.27	0.124	(−14.74, 73.66)
Procalcitonin (ug/L)	3.85 ± 7.49	3.44 ± 6.48	4.22 ± 8.36	0.197	(−2.54, 4.09)
IL-6 (pg/ml)	292.62 ± 695.96	255.82 ± 551.43	326.79 ± 812.90	0.582	(−238.62, 380.56)
Lactic acid (mmol/L)	2.45 ± 2.26	3.05 ± 3.06	1.95 ± 1.07	0.500	(−2.10, −0.95)
D-dimer (mg/L)	7.41 ± 6.94	7.33 ± 7.02	7.49 ± 6.97	0.702	(−3.03, 3.34)
APTT (s)	33.61 ± 7.09	33.83 ± 6.37	33.45 ± 7.66	0.697	(−3.62, 2.86)
PT (s)	13.05 ± 3.09	12.94 ± 2.30	13.14 ± 3.63	0.484	(−1.19, 1.58)
**Concomitant medications during the observation period, no. (%)**					
Norepinephrine	48 (57.83)	23 (60.53)	25 (55.56)	0.843	0.96 (0.67–1.39)
Midazolam	59 (71.08)	28 (73.68)	31 (68.89)	0.638	1.28 (0.82–1.27)
Propofol	72 (86.75)	35 (92.10)	37 (82.22)	0.269	0.91 (0.78–1.07)
Dexmedetomidine	61 (73.49)	28 (73.68)	33 (73.33)	0.892	1.02 (0.78–1.31)
Remifentanil	58 (69.88)	29 (76.31)	29 (64.44)	0.377	0.88 (0.67–1.16)
Sufentanyl	40 (48.19)	16 (42.11)	24 (53.33)	0.261	1.29 (0.82–2.05)
Fentanyl	12 (14.45)	4 (10.53)	8 (17.77)	0.328	1.72 (0.56–5.28)
Length of ICU stay, mean ± SD, day	20.14 ± 11.77	21.67 ± 13.14	19.02 ± 11.01	0.324	(−7.95, 2.66)
Length of hospital stay, mean ± SD, day	31.61 ± 22.21	34.48 ± 20.68	34.91 ± 27.13	0.938	(−10.36, 11.20)
28d mortality (%)	17 (20.48)	4 (10.53)	13 (28.89)	0.101	2.88 (0.80, 8.11)
In-hospital mortality (%)	25 (30.12)	11 (28.95)	14 (31.11)	0.918	0.95 (0.38, 2.41)

### 3.2. Gastric antrum echodensity measurement between effective and ineffective groups

**Figure 2:**
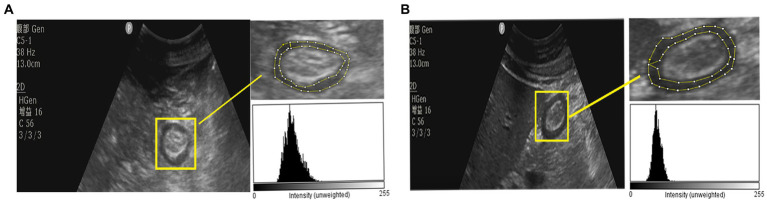
The POCUS image of gastric antrum echodensity between effective group and ineffective group. **(A)** The gastric antrum ultrasound image of a patient which was in ineffective group. **(B)** The gastric antrum ultrasound image of a patient which was in effective group.

**Figure 3:**
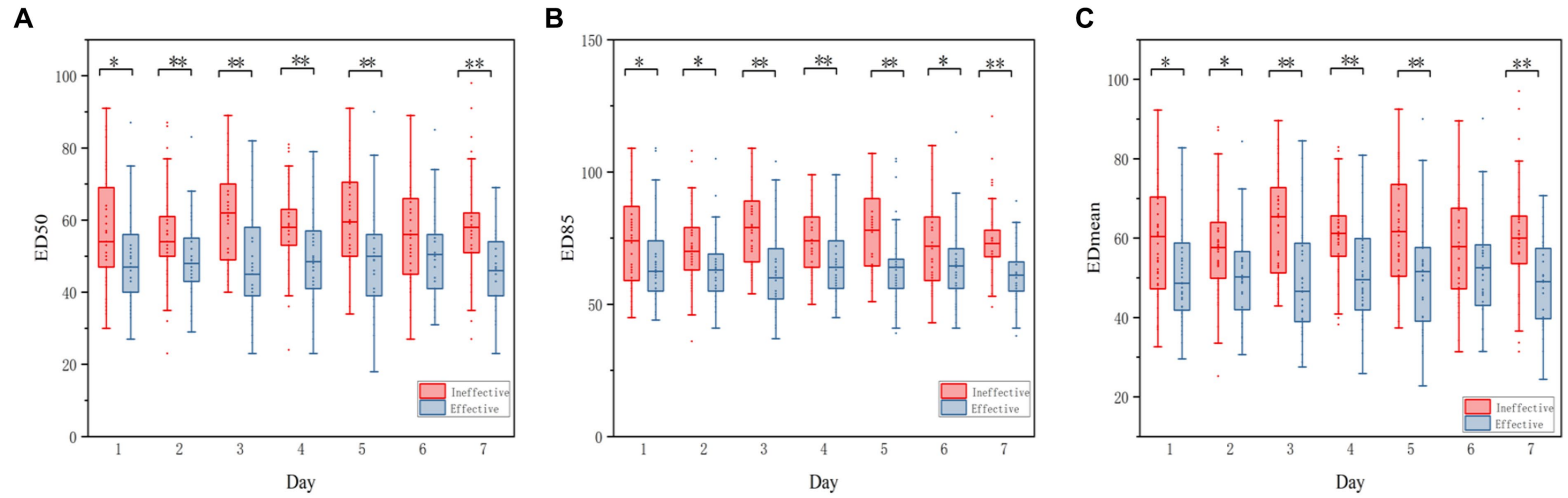
Boxplot of the change of ED_50_, ED_85_, ED_mean_ between effective group and Ineffective GROUP. Echodensity of gastric antrum between effective group and ineffective group before using the agents and until the seventh days. **(A)** Differences in ED_50_ between effective group and ineffective group; **(B)** Differences in ED_85_ between effective group and ineffective group; **(C)** Differences in ED_mean_ between effective group and ineffective group. The symbol of (*) represents *p* < 0.05, whereas the symbol of (**) represents *p* < 0.001 for all figures.

The echodensity of gastric antrum was assessed using the above-described protocol in accordance with our previous study. Point-of-care ultrasonography images of the gastric antrum echodensity in the effective and ineffective groups are shown in Figure 2. The mean difference of ED50, ED85 and ED_mean_ were 49.88 ± 13.78 vs. 58.13 ± 14.48 (*p* < 0.001), 65.70 ± 16.05 vs. 74.81 ± 16.41 (p < 0.001), 51.76 ± 14.07 vs. 60.18 ± 14.31 (*p* < 0.001) for effective group and ineffective group during the observational period, respectively. The gastric echodensity on the first to third days was significantly different between the effective and ineffective groups (Figure 3).

**Figure 4:**
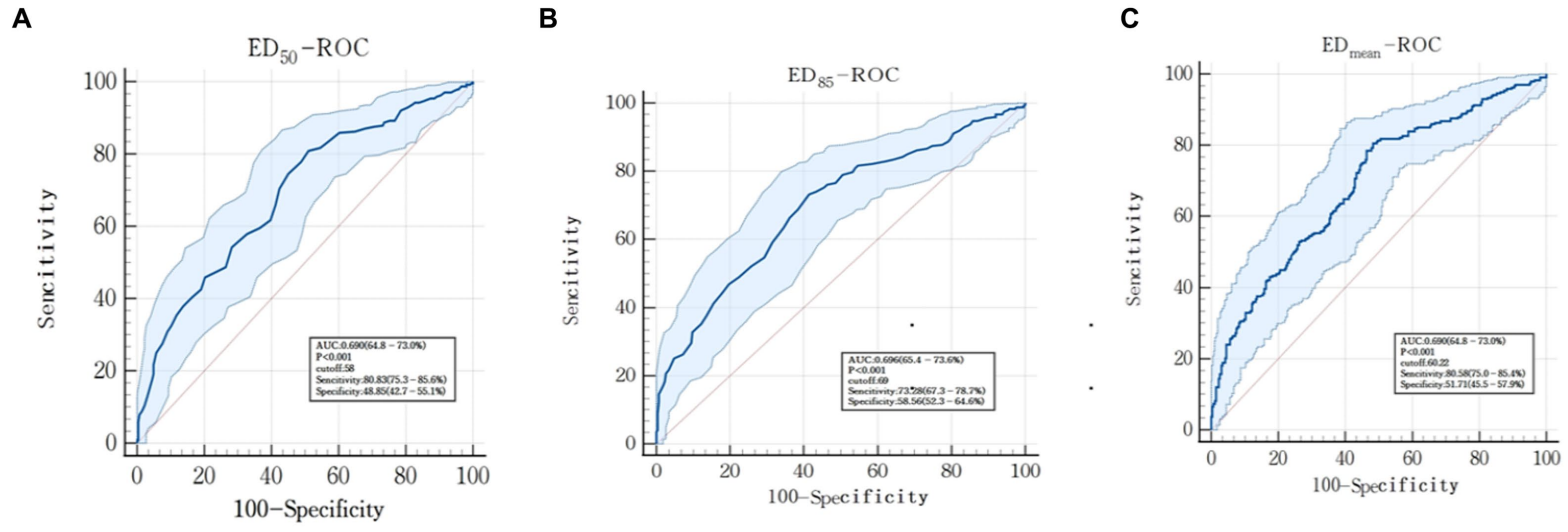
Receiver operator curve (ROC) to assess the ability of gastric antrum echodensity to evaluate the efficacy of prokinetic agents. **(A)** represents ED_50_-ROC; **(B)** represents ED_85_-ROC; **(C)** represents ED_mean_-ROC.

The threshold of the ED_50_ to distinguish effective from ineffective prokinetics was 58 with specificity of 48.85% [95% confidence interval (CI), 42.7–55.1%] and sensitivity of 80.83% (95% CI, 75.3–86.6%), and the ROC was 0.690 (95% CI, 64.8–73.0%; *p* < 0.001) (Figure 4A). The threshold of the ED_85_ to distinguish effective from ineffective prokinetics was 69 with specificity of 58.56% (95% CI, 52.3–64.6%) and sensitivity of 73.28% (95% CI, 67.3–78.7%), and the ROC was 0.696 (95% CI, 65.4–73.6%, *p* < 0.001) (Figure 4B). The threshold of the ED_mean_ to distinguish effective from ineffective prokinetics was 60.22 with specificity of 51.71% (95% CI, 45.5–57.9%) and sensitivity of 80.58% (95% CI, 75.0–85.4%), and the ROC was 0.690 (95% CI, 64.8–73.0%, *p* < 0.001) (Figure 4C).

The mean differences in the ED_50_, ED_85_, and ED_mean_ before using prokinetic agents are shown in Table 2. The mean duration of using prokinetic agents was 9.97 ± 6.21 and 8.76 ± 6.38 days (*p* = 0.420) in the effective group and ineffective group, respectively (Table 2). When feeding intolerance symptoms were persistent, placement of a nasointestinal tube was considered even when using prokinetic agents; a tube was placed in 19 (42.22%) patients in the ineffective group (Table 2).

**Table 2:** The association of ED_50_, ED_85_, ED_mean_ between effective group and ineffective group before using the prokinetic agents.

	Total (*n* = 83)	Effective group (*n* = 38)	Ineffective group (*n* = 45)	*p* value	Difference 95% CI
**Ultrasonic parameters before using the agents, mean ± SD**					
ED_50_	53.67 ± 13.92	48.78 ± 12.48	58.05 ± 13.72	<0.001	(−11.46, −7.05)
ED_85_	69.74 ± 15.86	64.15 ± 14.48	74.74 ± 15.37	<0.001	(−13.08, −8.09)
ED_mean_	55.71 ± 14.02	50.75 ± 12.87	60.15 ± 13.53	<0.001	(−11.59, −7.17)
The days of prokinetic agents	9.33 ± 6.29	9.97 ± 6.21	8.76 ± 6.38	0.420	(−1.76, 4.18)
The rate of placing nasointestinal tube after using the agents, No. (%)			19 (42.22)		

The differences in the AGI grade, GIDS, and GIF score were also compared between the two groups. A positive correlation was found between the gastric antrum echodensity and AGI (grade II or III) (*p* < 0.05) (Supplementary Figure S1). A positive correlation was also found between the gastric antrum echodensity and the GIDS (1 or 2) (*p* < 0.05) (Supplementary Figure S2) and the GIF score (1 or 2) (*p* < 0.05) (Supplementary Figure S3).

Furthermore, there were significant differences in the daily energy intake, daily protein intake, and daily volume of enteral nutrition during the observational period between the two groups (Supplementary Figure S4). The overall mean energy intake, protein intake, and volume of enteral nutrition for the 7-day period were 800.68 ± 449.78 kcal, 35.40 ± 21.66 g, and 791.87 ± 403.69 mL, respectively. The mean energy intake, protein intake, and volume of enteral nutrition in the effective vs. ineffective groups for the 7-day period were 16.21 ± 7.98 kcal/kg vs. 9.17 ± 6.43 kcal/kg (*p* < 0.001), 0.72 ± 0.38 g/kg vs. 0.42 ± 0.31 g/kg (*p* < 0.001), respectively.

**Figure 5:**
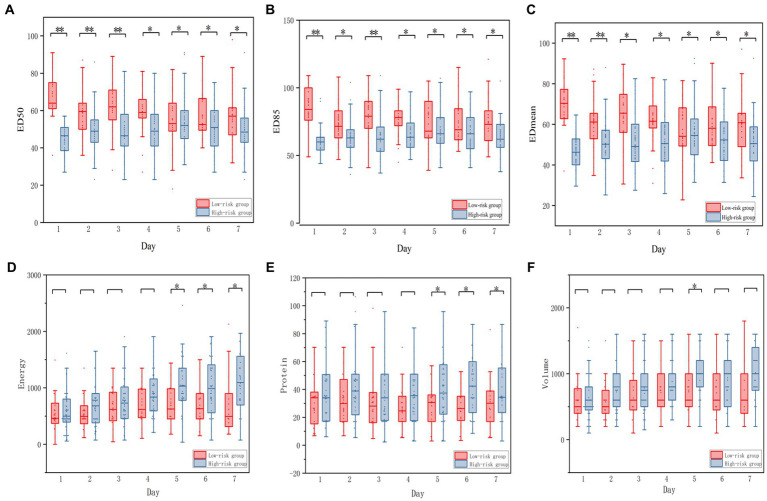
Boxplot of the daily energy, daily protein, daily volume of enteral nutrition between low-risk and high-risk group. **(A–C)** Representing the ED_50_, ED_85_, ED_mean_ between low-risk and High-risk group, respectively. **(D–F)** representing the energy, protein, volume between Low-risk and High-risk group respectively.

The optimal ED_50_ cutoff value for predicting the efficacy of prokinetic agents was 58. We then categorized the patients according to the ED_50_ of the first day, before using prokinetic agents: the high-risk group (ED_50_ > 58, *n* = 33) and the low-risk group (ED_50_ ≤ 58, *n* = 50). Among all parameters, only the lactic acid level was significantly different between the two groups. The ED_50_, ED_85_, ED_mean_, and the overall nutrition parameters during the observational period were significantly different between the groups, and their daily variations are shown in Figure 5. There was a significant between-group difference in the ratio of effectiveness of prokinetic agents (*n* = 32, 64.00% vs. *n* = 7, 21.21%; *p* < 0.001), but there was no significant difference in the incidence of placing a nasointestinal tube (*n* = 9, 18.00% vs. *n* = 10, 30.30%; *p* = 0.438). The baseline demographic information in the two groups is shown in Table 3.

**Table 3:** Baseline and characteristics of patients in prokinetic agents low-risk and high-risk group.

Characteristic	Total (*n* = 83)	Low-risk group (*n* = 50)	High-risk group (*n* = 33)	*p* value	OR or difference 95% CI
Age (years), mean ± SD	56.94 ± 16.61	54.8 ± 14.8	58.2 ± 18.1	0.363	(−13.37, 0.38)
Male sex, no. (%)	57 (68.67)	29 (58.00)	28 (84.84)	0.141	(−12.82, 3.58)
BMI, mean ± SD	23.53 ± 4.16	23.01 ± 4.59	24.12 ± 3.66	0.249	(−13.16, 0.67)
APHACHE II score, mean ± SD	17.29 ± 8.41	16.35 ± 7.87	18.78 ± 9.14	0.202	(−3.39, 1.98)
SOFA score, mean ± SD	9.59 ± 3.79	9.4 ± 3.8	10.3 ± 3.5	0.308	(−13.37, 0.38)
**Laboratory tests at admission, mean ± SD**					
White blood cell (*10^9^/L)	11.10 ± 6.89	11.42 ± 7.11	12.22 ± 6.62	0.448	(−13.16, 0.67)
Hemoglobin (g/L)	99.07 ± 36.65	98.20 ± 23.88	100.47 ± 51.29	0.945	(−3.39, 1.98)
Platelet (*10^9^/L)	172.2 ± 106.4	174.83 ± 101.94	168.82 ± 114.84	0.352	(−13.37, 0.38)
Albumin (g/L)	32.62 ± 5.71	32.52 ± 6.08	32.78 ± 5.14	0.849	(−12.82, 3.58)
Total bilirubin (umol/L)	32.62 ± 5.71	32.79 ± 5.73	32.53 ± 5.93	0.419	(−13.16, 0.67)
Serum creatinine (umol/L)	128.56 ± 128.12	114.39 ± 108.21	123.34 ± 138.95	0.757	(−3.39, 1.98)
Glucose (mmol/L)	11.19 ± 6.60	10.73 ± 5.68	11.92 ± 7.79	0.500	(−13.37, 0.38)
C-reactive protein (mg/L)	114.08 ± 101.60	110.43 ± 91.58	120.10 ± 117.63	0.502	(−12.82, 3.58)
Procalcitonin (ug/L)	5.50 ± 16.66	3.86 ± 7.21	8.21 ± 25.51	0.529	(−13.16, 0.67)
IL-6 (pg/ml)	292.10 ± 696.17	343.94 ± 824.70	203.97 ± 389.34	0.052	(−3.39, 1.98)
Lactic acid (mmol/L)	2.45 ± 2.27	2.82 ± 2.77	1.86 ± 0.82	0.009	(−13.37, 0.38)
D-dimer (mg/L)	7.47 ± 6.97	7.06 ± 5.88	7.52 ± 7.81	0.780	(−12.82, 3.58)
APTT (s)	33.63 ± 7.13	33.51 ± 6.71	33.50 ± 7.62	0.993	(−13.16, 0.67)
PT (s)	13.06 ± 3.10	13.08 ± 3.46	12.97 ± 2.98	0.900	(−3.39, 1.98)
Length of ICU stay, mean ± SD, day	20.25 ± 12.04	20.25 ± 11.20	20.25 ± 13.43	0.999	(−13.37, 0.38)
Length of hospital stay, mean ± SD, day	33.20 ± 21.23	33.12 ± 19.37	33.33 ± 24.32	0.966	(−12.82, 3.58)
28d mortality (%)	17 (20.48)	9 (18.00)	8 (24.24)	0.377	(−13.16, 0.67)
In-hospital mortality (%)	25 (30.12)	15 (30.00)	10 (30.30)	0.801	(−3.39, 1.98)
**Ultrasonic parameters during the observational period, mean ± SD**					
ED_50_	53.94 ± 14.65	49.51 ± 12.89	62.72 ± 14.08	<0.001	(−12.82, 3.58)
ED_85_	70.19 ± 16.65	65.13 ± 14.26	80.15 ± 16.91	<0.001	(−13.16, 0.67)
ED_mean_	55.87 ± 14.68	51.44 ± 12.98	64.75 ± 14.04	<0.001	(−3.39, 1.98)
**Nutrition during the observational period, mean ± SD**					
Energy	803.04 ± 452.51	838.68 ± 466.61	641.21 ± 364.64	<0.001	(−12.82, 3.58)
Protein	35.32 ± 21.62	37.19 ± 22.77	27.73 ± 16.61	<0.001	(−13.16, 0.67)
Volume	798.86 ± 400.69	836.26 ± 391.04	659.42 ± 361.04	<0.001	(−3.39, 1.98)
The efficacy of prokinetic agents, no. (%)	39 (46.99)	32 (64.00)	7 (21.21)	<0.001	(−13.37, 0.38)
The days of prokinetic agents	8.94 ± 6.09	9.31 ± 6.10	8.60 ± 6.13	0.605	(−12.82, 3.58)
The rate of placing nasointestinal tube after using the agents, no. (%)	19 (22.89)	9 (18.00)	10 (30.30)	0.438	(−13.16, 0.67)

In the univariate logistic regression analysis, the following were associated with the efficacy of prokinetic agents: ED_50_ [odds ratio (OR), 1.051; 95% CI, 1.036–1.066], ED_85_ (OR, 1.043; 95% CI, 1.030–1.056), ED_mean_ (OR, 1.050; 95% CI, 1.036–1.065), and age (OR, 1.017; 95% CI, 1.006–1.028). The univariate logistic regression analysis also showed that sepsis and cardiovascular disease as reasons for admission to the ICU, the use of sufentanil and fentanyl during the observational period, and the levels of serum creatinine, PCO_2_, and sodium obtained at ICU admission were also associated with prokinetic agent efficacy. In the multivariable logistic regression analysis of prokinetic agent efficacy, a higher gastric antrum echodensity was more likely to be associated with ineffectiveness. The ORs of the ED_50_, ED_85_, and ED_mean_ in Models 1 to 3 were as follows. Model 1: ED_50_ (OR, 1.059; 95% CI, 1.043–1.076; *p* < 0.001), ED_85_ (OR, 1.041; 95% CI, 1.028–1.055; *p* < 0.001), and ED_mean_ (OR, 1.048; 95% CI, 1.033–1.064; *p* < 0.001). Model 2: ED_50_ (OR, 1.049; 95% CI, 1.032–1.066; *p* < 0.001), ED_85_ (OR, 1.043; 95% CI, 1.029–1.057; *p* < 0.001), and ED_mean_ (OR, 1.051; 95% CI, 1.035–1.067; *p* < 0.001). Model 3: ED_50_ (OR, 1.049; 95% CI, 1.032–1.067; *p* < 0.001), ED_85_ (OR, 1.042; 95% CI, 1.027–1.057; *p* < 0.001), and ED_mean_ (OR, 1.050; 95% CI, 1.032–1.067; *p* < 0.001). The other variables are shown in Table 4. And the logistic regression analysis of ED_85_ and ED_mean_ were shown in Supplementary Tables S1, S2.

**Table 4:** The logistic regression analysis to test the efficacy of prokinetic agents with ED_50,_ ED_85_, and ED_mean_.

Variable	Unadjusted OR (95% CI)	Unadjusted *p* value	Model 1		Model 2		Model 3	
			Odds ratio (95% CI)	*p* value	Odds ratio (95% CI)	*p* value	Odds ratio (95% CI)	*p* value
ED_50_	1.051 (1.036–1.066)	<0.001	1.059 (1.043–1.076)	<0.001	1.409 (1.032–1.066)	<0.001	1.049 (1.032–1.067)	<0.001
ED_85_	1.043 (1.030–1.056)	<0.001	1.041 (1.028–1.055)	<0.001	1.043 (1.029–1.057)	<0.001	1.042 (1.027–1.057)	<0.001
ED_mean_	1.050 (1.036–1.065)	<0.001	1.048 (1.033–1.064)	<0.001	1.051 (1.035–1.067)	<0.001	1.050 (1.032–1.067)	<0.001
Age	1.017 (1.006–1.028)	0.002	0.993 (0.980–1.006)	0.293	0.984 (0.969–0.999)	0.042	0.992 (0.976–1.007)	0.292
Sepsis	4.579 (2.692–7.790)	<0.001	5.249 (2.879–9.570)	<0.001	9.516 (4.904–18.462)	<0.001	4.842 (2.246–9.665)	<0.001
Cardiovascular diseases	2.406 (1.340–4.317)	0.003	3.359 (1.798–6.276)	0.003	3.859 (2.025–7.357)	<0.001	3.639 (1.869–7.296)	<0.001
Sufentanyl	1.445 (1.102–2.063)	0.043			4.001 (2.197–7.287)	0.001	2.313 (1.479–3.619)	<0.001
Fentanyl	2.509 (1.456–4.324)	0.001			2.022 (1.327–3.083)	<0.001	4.121 (2.190–7.755)	<0.001
Serum creatinine	1.004 (1.002–1.006)	<0.001					1.002 (1.000–1.004)	0.092
PCO2	1.063 (1.040–1.087)	<0.001					1.041 (1.016–1.067)	0.001
Sodium	1.076 (1.043–1.110)	<0.001					1.071 (1.032–1.112)	<0.001

Model 1 was adjusted for age, Sepsis, Cardiovascular Diseases. Model 2 was adjusted for age, Sepsis, Cardiovascular Diseases, Sufentanyl and Fentanyl. Model 3 was adjusted for age, Sepsis, Cardiovascular Diseases, Sufentanyl and Fentanyl, Serum creatinine, PCO2 and Sodium.

There is collinearity among ED_50_, ED_85_, and ED_mean_ and cannot be included in the same model. In Model 1, 2, and 3, the results for these variables were generated by only entering the ED_50_ into the multivariable-adjusted model. Among them, Sepsis and Cardiovascular Diseases were the reason for admission to ICU; Sufentanyl and Fentanyl were the frequency of patients during the observational period; the level of Serum creatinine, PCO2 and Sodium were obtained at ICU admission.

Considering that several different prokinetic agents were used in this cohort, a subgroup analysis of the type of prokinetic agents was performed. Supplementary Figures S5–S10 show the mean differences in the ED_50_, ED_85_, and ED_mean_ between the ineffective and effective groups according to subgroups of prokinetic agents. The energy intake, protein intake, and volume of enteral nutrition are also shown in Supplementary Figures S5–S10. The baseline characteristics are compared between the two groups in Supplementary Tables S3–S8.

The analysis of ROC for these risk factors such as sepsis, cardiovascular diseases were evaluated and showed in Supplementary Figure S11. There was no difference in Cardiovascular Diseases, Sufentanyl and Fentanyl in the analysis. However, some difference was existed in the level of sodium and PCO_2_ and the sensitivity of ED_50_, ED_85_, and ED_mean_ were prior compared with sodium and PCO_2_ with lower specificity than these parameters.

## 4. Discussion

In this study, we evaluated the ability of the gastric antrum echodensity to predict the efficacy of prokinetic agents in critically ill patients who develop feeding intolerance during enteral feeding. Our results showed significant differences in the gastric antrum echodensity between the effective and ineffective groups, especially on the first day before treatment with prokinetic agents was begun. This finding indicates that the gastric antrum echodensity might serve as a useful decision-making tool for clinicians managing patients with EFI. This study showed that the echodensity of the gastric antrum may help predict the efficacy of prokinetic agents in critically ill patients with EFI. Furthermore, our study showed that the proportion of patients requiring nasointestinal tube placement was nearly halved in the ineffective group, providing insight into the optimal enteral pathway to consider when initiating enteral feeding. Overall, our findings suggest that the gastric antrum echodensity may be a useful tool in predicting the efficacy of prokinetic agents for critically ill patients with feeding intolerance.

Enteral feeding intolerance is associated with poor outcomes, such as prolongation of the ICU stay and increased mortality, compared with patients who tolerate enteral feeding. EFI is mainly caused by gastrointestinal dysmotility, which leads to slow gastric emptying and a high GRV. More than 50% of patients in the ICU develop EFI, which is associated with increased mortality in these patients. Such patients require intervention with treatment measures such as prokinetic agents (12, 27).

Some recent studies have shown that ultrasonography is a potentially useful tool in evaluating gastrointestinal dysfunction in critically ill patients. Acute increases in muscle sonographic echodensity can reportedly reflect muscle injury at the cellular level. Additionally, Coiffard et al. reported that increased diaphragm echodensity during the early course of mechanical ventilation was associated with prolonged mechanical ventilation (28–30). Our previous study also showed that the gastric antrum echodensity was associated with the grade of AGI and feeding intolerance (19). Therefore, as we begun the present study, we assumed that ultrasound assessment of the echodensity of the gastric antrum could be a useful tool to evaluate the efficacy of prokinetic agents.

We performed a subgroup analysis of different types of prokinetic agents, and there was a significant difference between the effective group and ineffective group in all treatment regimens except two: use of domperidone and use of the combination of metoclopramide, domperidone, and mosapride. This result might have been caused by the small sample of patients. This could also suggest that the echodensity of the gastric antrum can assist in evaluating the effectiveness of prokinetic agents in critically ill patients.

Previous guidelines have recommended 60% as the threshold for reaching the target doses of enteral nutrition (10) with a little difference in our study. A recent study used a percentage daily protein prescription of >80% to distinguish the efficacy of ulimorelin and metoclopramide. Arabi et al. found that the caloric goal of 40–60% of total calories was not associated with lower mortality compared with planned delivery of the full amounts of calories (13, 31). In the present study, the daily energy and protein intake was collected in the first 7 days after initiating treatment with prokinetic agents, and an energy to target energy ratio of >50% at 7 days indicated that the prokinetic agents were effective. The interobserver consistency was not assessed because the investigators showed high consistency in their assessment of echodensity in our previous study. The types of some prokinetic agents were not consistent with the guidelines’ recommendations, but relevant studies showed that domperidone can be used to manage EFI and mosapride could improve gastric emptying (26, 32, 33).

The rate of diagnosis of diabetes was approximately twice as high in the ineffective group but no statistical difference was found between the group. Moreover, in the *post hoc* analysis, there was no significant heterogeneity in the effect between the subgroups (age > 65y or < =65y, gender, comorbidity of diabetes and hypertension, SOFA scores > = 10 or < 10, APACHE II scores > = 20 or < 20). And relative studies show that the association between gastrointestinal symptoms and autonomic or peripheral neuropathy is relatively weak (34, 35).

The ED_50_ threshold calculated from the theses cases was used to differentiate between the low-risk and high-risk groups. While the validation cohort which should exclude the initial cases needs to be verified in another cohort. The purpose of the analysis was to show whether there were potential differences in baseline characteristics in the low-risk and high-risk groups and to explore the feasibility of gastric antrum echodensity and further studies will be performed in the future to extrapolate the results.

A related study showed that excessive fluid resuscitation and gastrointestinal tissue edema could affect gastrointestinal motility (7), but there was no difference in the daily fluid volume between the two groups. Unfortunately, tissue edema was not monitored in this study. The increased intra-abdominal pressure on gastrointestinal intolerance could influence the efficacy of prokinetic drug therapy in patients with high intra-abdominal pressure. However, we generally monitor intra-abdominal pressure in severe acute pancreatitis patients which were initially excluded from the study. One of the main reasons for feeding intolerance was GI dysmotility, and the prevalence of GI motility disorders reached as high as 70%. Although we designed the study for feeding intolerance in critically ill patients, only gastric function was evaluated in our study, and intestinal function should be considered in further studies.

This is the first study to describe a novel technique for assessment of the efficacy of prokinetic agents in critically patients with EFI. We identified the percentile grayscale values (ED_50_, ED_85_, and ED_mean_) of the echodensity to indicate the differences in prokinetic agents between the ineffective and effective groups. However, this study had some limitations. First, it was a single-center prospective study with a small sample size; this could limit the ability to extrapolate the results, cause low statistical power, and increase the risk of type II errors. Thus, our findings require validation in external cohorts from larger samples. Second, we only used a Philips ultrasound device; this may prevent generalization of our findings to other ultrasound devices or parameter settings. Third, we did not assess the potential risks and adverse effects of using prokinetic agents. Fourth, the results of the study are limited by the exclusion of patients who underwent abdominal surgery and by the requirement for extra time and specialized software to process the echodensity data. Finally, is also important to note that the use of some prokinetic agents in this study may not align with current guidelines and that ultrasound imaging may not be feasible in patients with intestinal pneumatosis or significant abdominal distension.

## 5. Conclusion

Evaluation of the gastric antrum echodensity could prove to be a valuable tool in determining the efficacy of prokinetic agents. Prokinetic agents are more likely to be ineffective in patients with increased gastric antrum echodensity. Gastric antrum echodensity might help clinicians decide whether to use prokinetic agents or place a post-pyloric tube when EFI occurs in critically ill patients.

## Data availability statement

The original contributions presented in the study are included in the article/Supplementary material, further inquiries can be directed to the corresponding author.

## Ethics statement

The study protocol was approved by the West China Hospital of Sichuan University Biomedical Research Ethics Committee (No. 2022S424). The informed consents of each patient were obtained from patients or their next-of-kin.

## Author contributions

QW and GL contributed to study design. XF, YW, LW, HF, and XX contributed to data acquisition. GL, JY, and XF study analysis and interpretation of data. BW, ZZ, XJ, and YC contributed to recorded and checked data. YK, HY, and YC contributed to revise the manuscript for important intellectual content. GL, YC, and QW wrote the manuscript. QW substantively revised the manuscript. All authors contributed to the article and approved the submitted version.

## Figures and Tables

**Table:** 

EN	Enteral nutrition
EFI	Enteral feeding intolerance
GRV	Gastric residual volume
HGRV	High gastric residual volume
GUTS	Gastrointestinal and urinary tract sonography
AGI	Gastrointestinal injury
FI	Feeding intolerance
ICU	Intensive care unit
BMI	Body mass index
APACHE II	Acute physiology and chronic health evaluation
SOFA	Sequential organ failure assessment
APTT	Activated partial thromboplastin time
PT	Prothrombin time
GIF	Gastrointestinal failure
GIDS	Gastrointestinal dysfunction score
OR	Odds ratio
SD	Standard deviation
PCO2	Arterial carbon dioxide partial pressure
COPD	Chronic obstructive pulmonary disease
SLE	Systemic lupus erythematosus
CKD	Chronic kidney disease
