# Supplementary material for: Prediction of prokinetic agents in critically ill patients with feeding intolerance: a prospective observational clinical study
Source: Front Nutr. 2023 Oct 27;10:1244517. doi: 10.3389/fnut.2023.1244517 (PMC10641452; doi:10.3389/fnut.2023.1244517)
Supplement: Supplementary file 1 [file Data_Sheet_1.docx]

**Supplementary Material**

Prediction of Prokinetic Agents in Critically Ill Patients with Feeding Intolerance: A Prospective Observational Clinical Study

Guangxuan Lv, MD1; Tao Zhang, MD1; Luping Wang, MD1; Xin Fu, MD1; Yucong Wang, MD1; Hua Yao, MD1; Huan Fang, MD1; Xiaoxiao Xia, MD1; Jing Yang, MD1; Bo Wang, MD1; Zhongwei Zhang, MD1; Xiaodong Jin, MD1; Yan Kang, MD1; Yisong Cheng, MD1*; Qin Wu, MD1*

Department of Critical Care Medicine, West China Hospital, Sichuan University, Chengdu, China.

**Correspondence:**

Qin Wu

[Qinwu0221@gmail.com](mailto:Qinwu0221@gmail.com)

Yisong Cheng,

yisongcheng01@163.com

Email addresses:

GL: 18846054403@163.com

TZ: zt921013y@163.com

LW: eeeeewlp@163.com

XF: fffuxin1999@163.com

YW: wangyucong0818@qq.com

HY: [yaohua0312@126.com](mailto:yaohua0312@126.com)

HF: 1766127084@qq.com

XX: 1247564590@qq.com

YJ: yjingscu@163.clm

BW: wchicu@126.com

ZZ: 716461751@qq.com

XJ: zh_jxd@163.com

YK: kangyan@scu.edu.cn

YC: yisongcheng01@163.com

QW: [Qinwu0221@gmail.com](mailto:Qinwu0221@gmail.com)

**Supplementary Fig. 1-Fig 3.** Boxplot of the change of ED_50_, ED_85_, ED_mean_ with AGI (Fig 1a.-c.), GIDS (Fig 2d.-f.) and GIF (Fig 3g.-i.) score between Effective group and Ineffective group. AGI score Acute Gastrointestinal Injury; GIDS score Gastrointestinal Dysfunction Score; GIF score Gastrointestinal Failure score

**Fig 1.** Echodensity change of gastric antrum (ED_50_, ED_85_, ED_mean_) between Effective group and Ineffective group with AGI (Fig 1a.-c.)


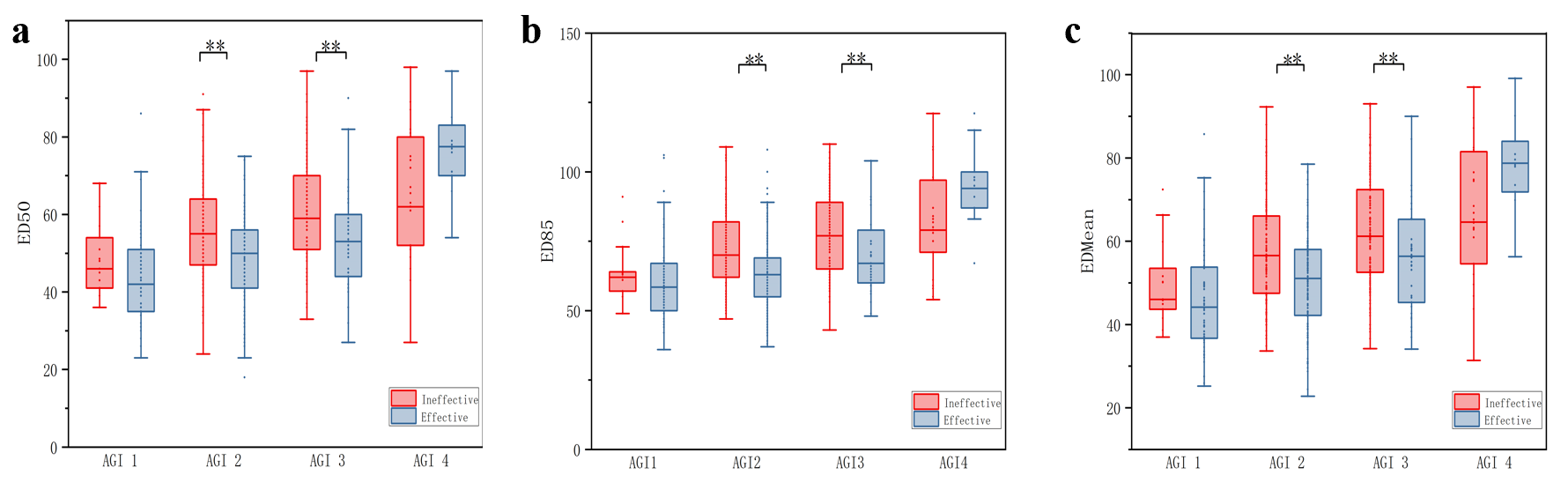


**Fig.2.** Echodensity change of gastric antrum (ED_50_, ED_85_, ED_mean_) between Effective group and Ineffective group with GIDS (Fig 2d.-f.)


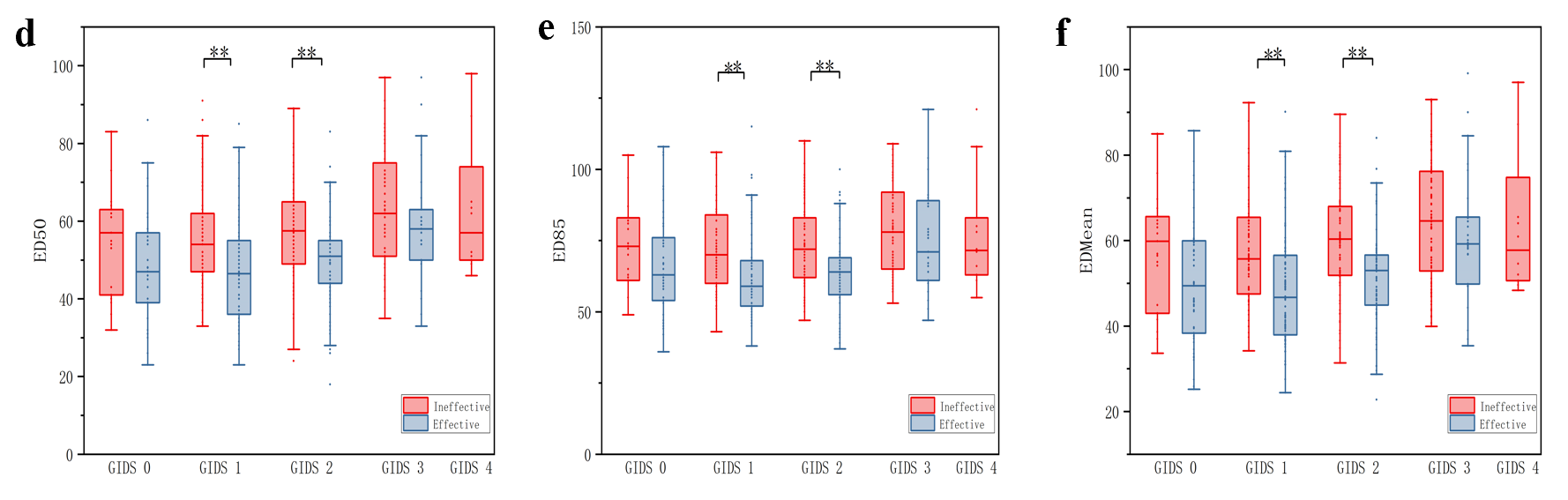


**Fig.3.** Echodensity change of gastric antrum (ED_50_, ED_85_, ED_mean_) between Effective group and Ineffective group with GIF (Fig 3g.-i.)


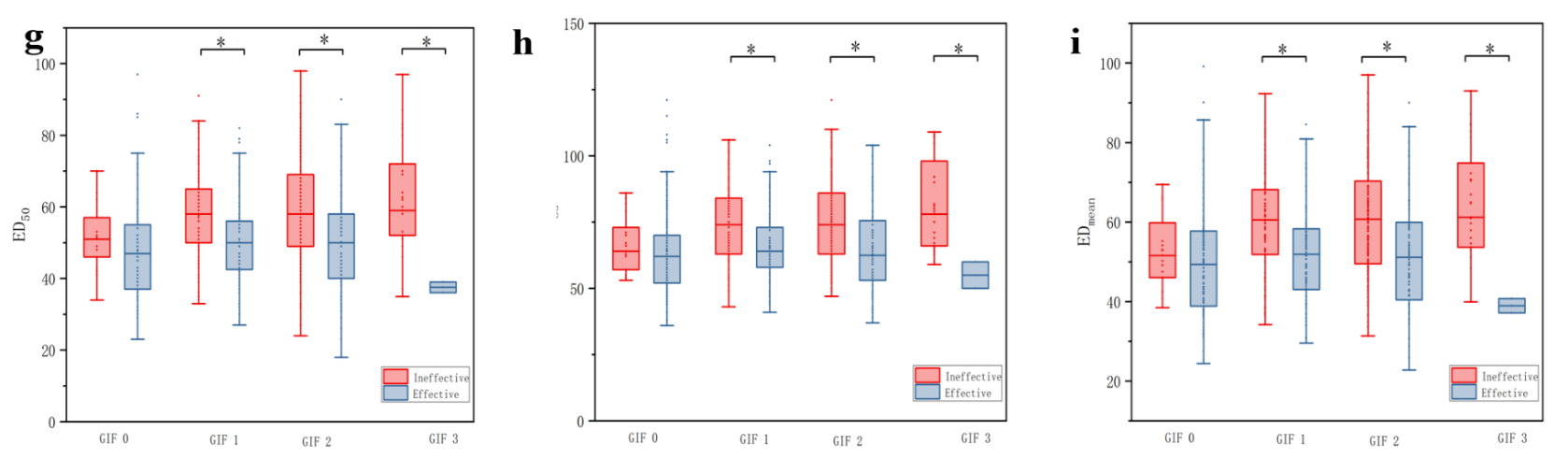


**Supplementary Fig.4.** Boxplot of the Daily Energy, Daily Protein, Daily Volume of enteral nutrition between Effective group and Ineffective group


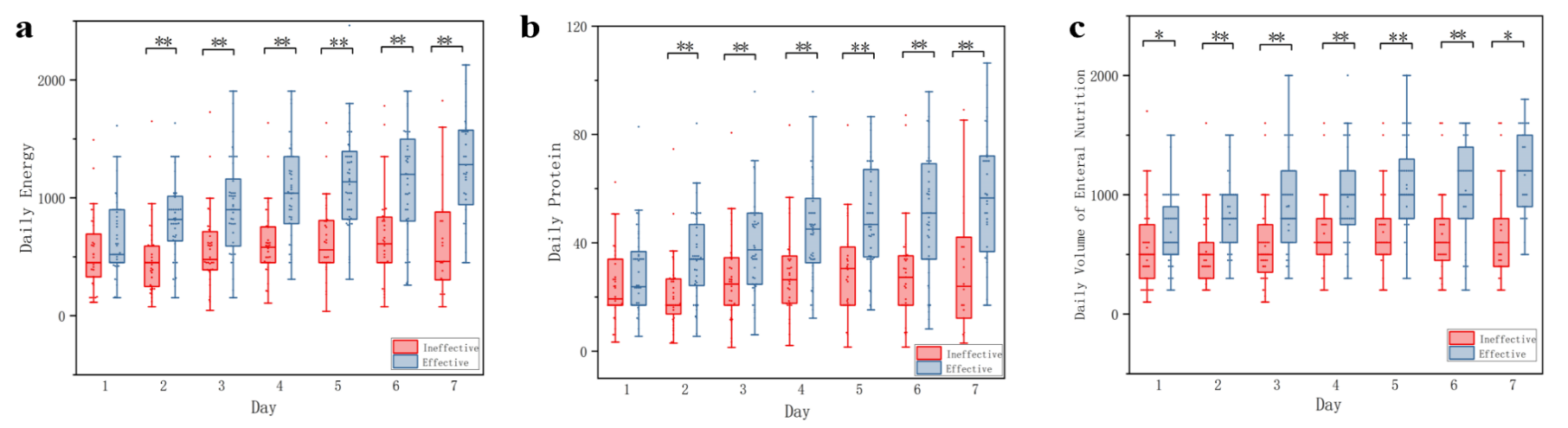


a, b and c representing the daily energy, daily protein, daily volume of enteral nutrition between Effective group and Ineffective group respectively.

**Supplementary Fig.5.** Boxplot of the ED_50_, ED_85_, ED_mean,_ Daily Energy, Daily Protein, Daily Volume of enteral nutrition between Effective group and Ineffective group using the metoclopramide agents.


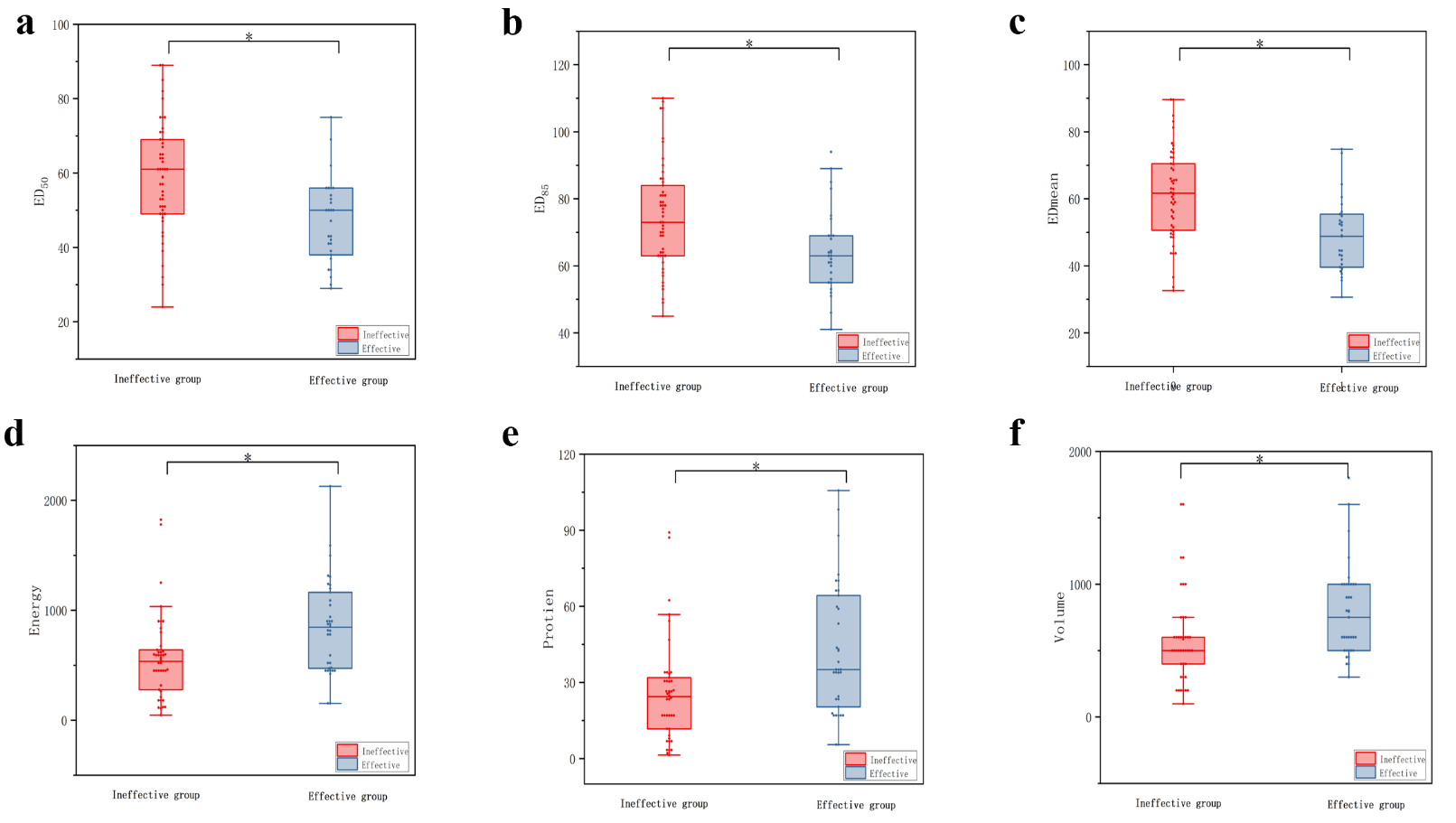


a, b and c representing the ED_50_, ED_85_, ED_mean_ between Effective group and Ineffective group using the metoclopramide agents respectively.

d, e and f representing the energy, protein and volume between Effective group and Ineffective group using the metoclopramide agents respectively.

**Supplementary Fig.6.** Boxplot of the ED_50_, ED_85_, ED_mean,_ Daily Energy, Daily Protein, Daily Volume of enteral nutrition between Effective group and Ineffective group using the mosapride agents.


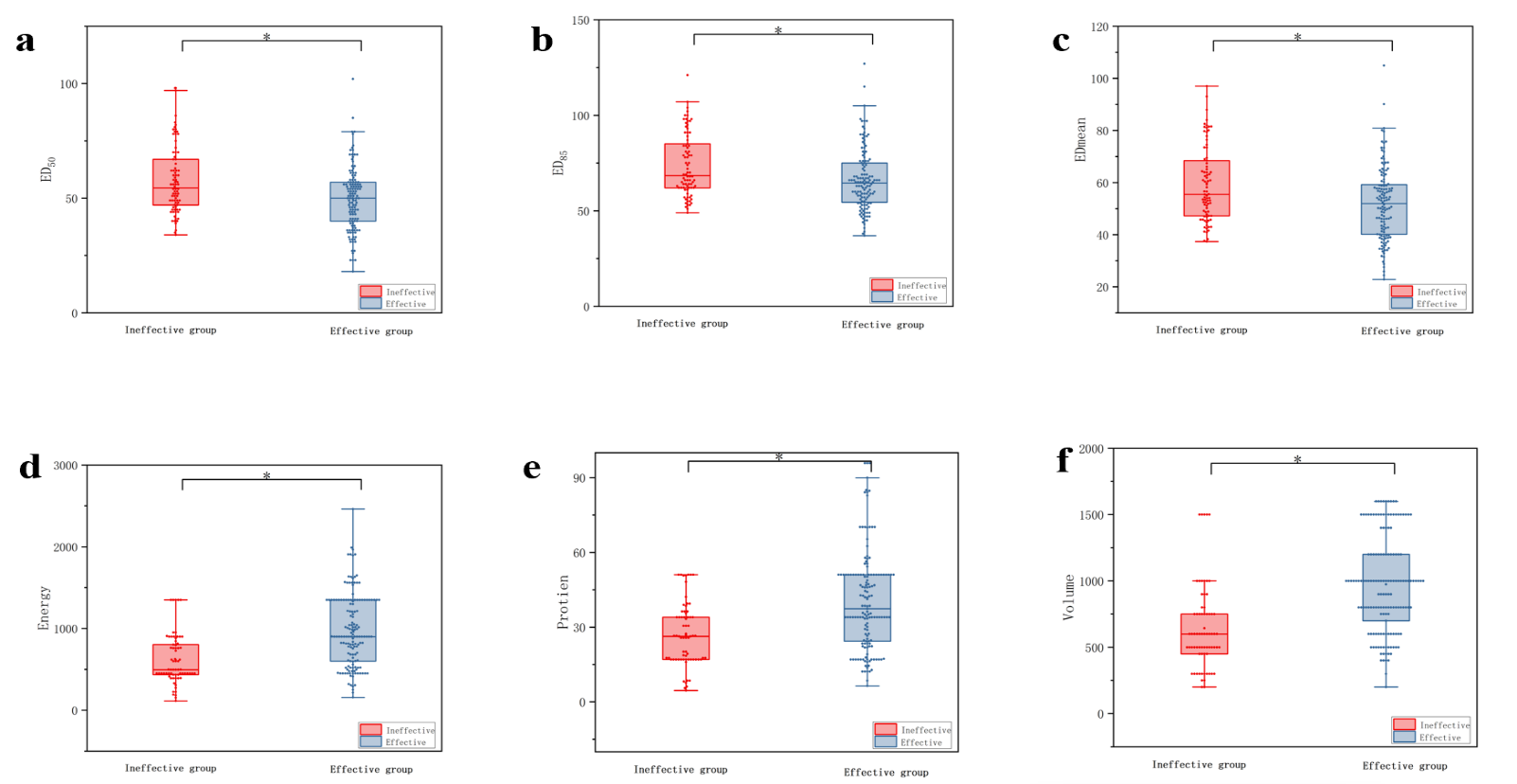


**a.**

**d.**

a, b and c representing the ED_50_, ED_85_, ED_mean_ between Effective group and Ineffective group using the mosapride agents respectively.

d, e and f representing the energy, protein and volume between Effective group and Ineffective group using the mosapride agents respectively.

**Supplementary Fig.7.** Boxplot of the ED_50_, ED_85_, ED_mean,_ Daily Energy, Daily Protein, Daily Volume of enteral nutrition between Effective group and Ineffective group using the domperidone and mosapride agents.


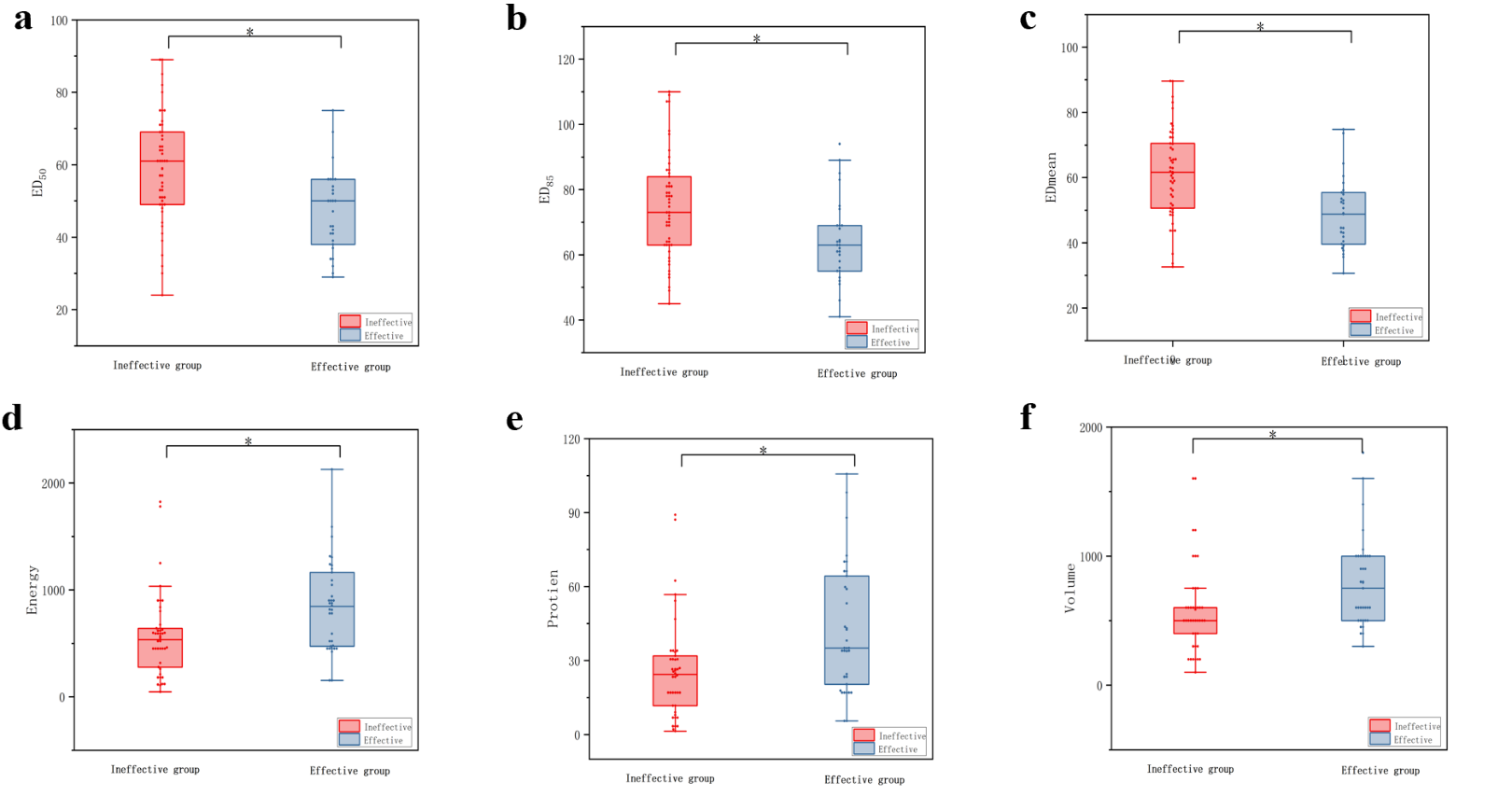


**a.**

**d.**

a, b and c representing the ED_50_, ED_85_, ED_mean_ between Effective group and Ineffective group using the domperidone and mosapride agents respectively.

d, e and f representing the energy, protein and volume between Effective group and Ineffective group using the domperidone and mosapride agents respectively.

**Supplementary Fig.8.** Boxplot of the ED_50_, ED_85_, ED_mean,_ Daily Energy, Daily Protein, Daily Volume of enteral nutrition between Effective group and Ineffective group using the metoclopramide and mosapride agents.


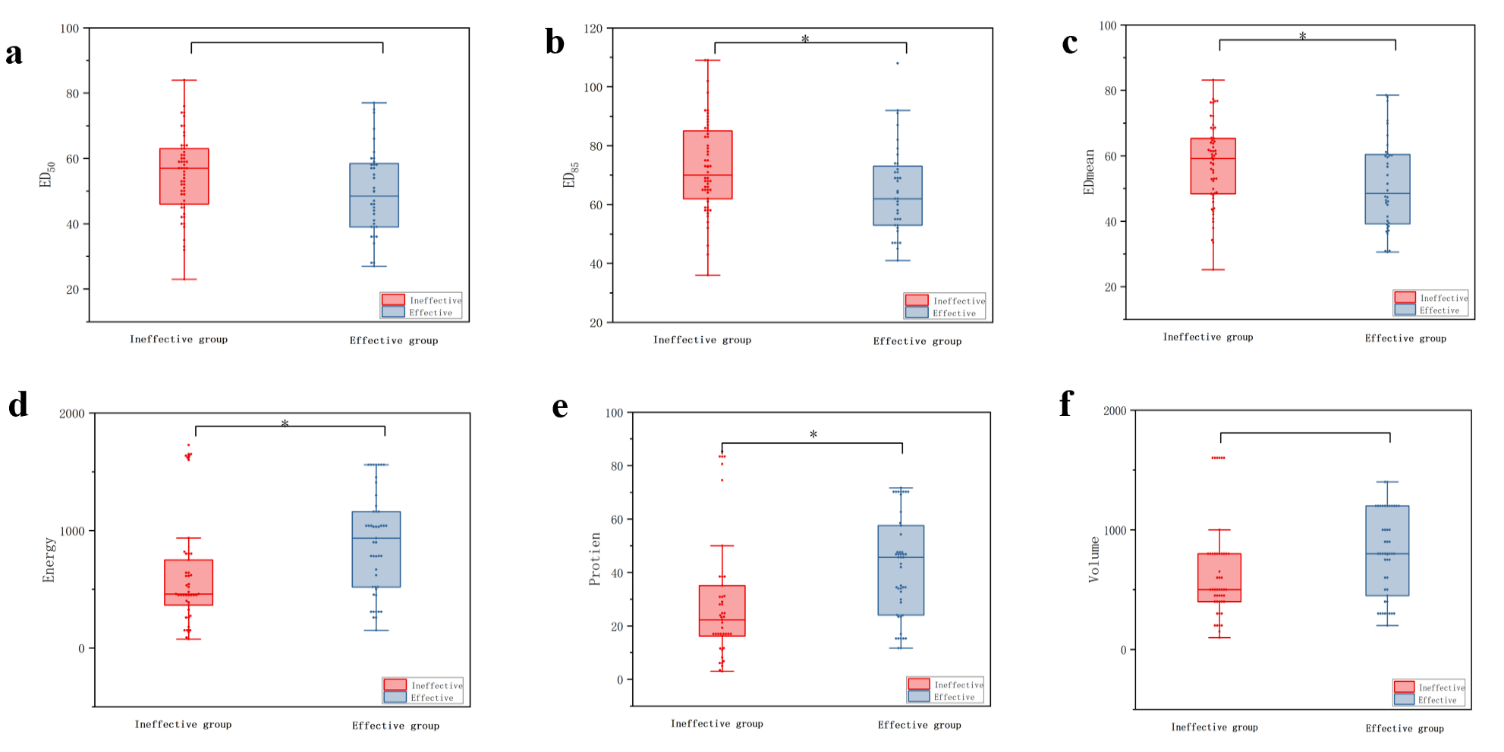


a, b and c representing the ED_50_, ED_85_, ED_mean_ between Effective group and Ineffective group using the metoclopramide and mosapride agents respectively.

d, e and f representing the energy, protein and volume between Effective group and Ineffective group using the domperidone and metoclopramide agents respectively.


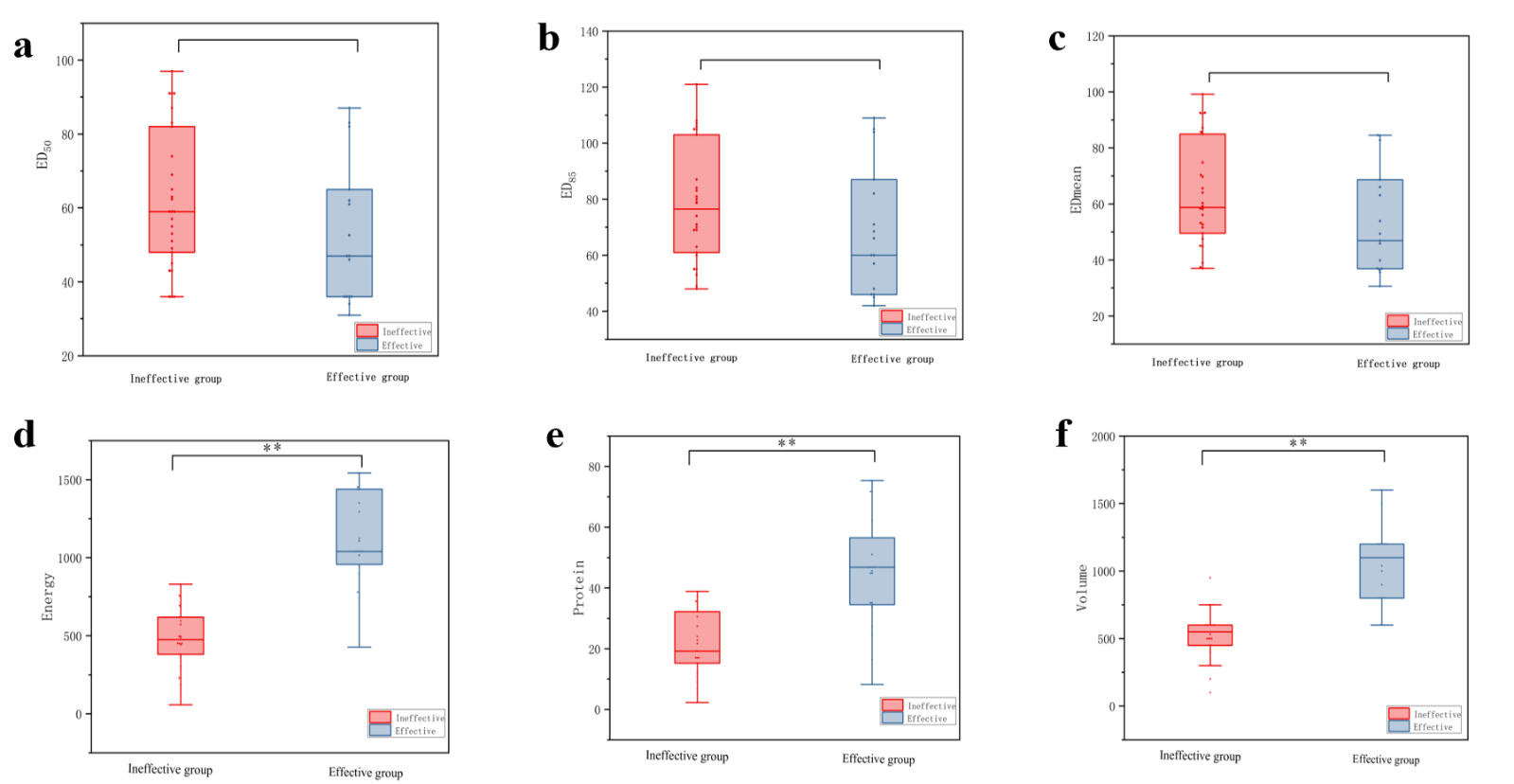
**Supplementary Fig.9.** Boxplot of the ED_50_, ED_85_, ED_mean,_ Daily Energy, Daily Protein, Daily Volume of enteral nutrition between Effective group and Ineffective group using the domperidone agents.

a, b and c representing the ED_50_, ED_85_, ED_mean_ between Effective group and Ineffective group using the domperidone agents respectively.

d, e and f representing the energy, protein and volume between Effective group and Ineffective group using the domperidone agents respectively.

**Supplementary Fig.10.** Boxplot of the ED_50_, ED_85_, ED_mean,_ Daily Energy, Daily Protein, Daily Volume of enteral nutrition between Effective group and Ineffective group using the metoclopramide, domperidone and mosapride agents.


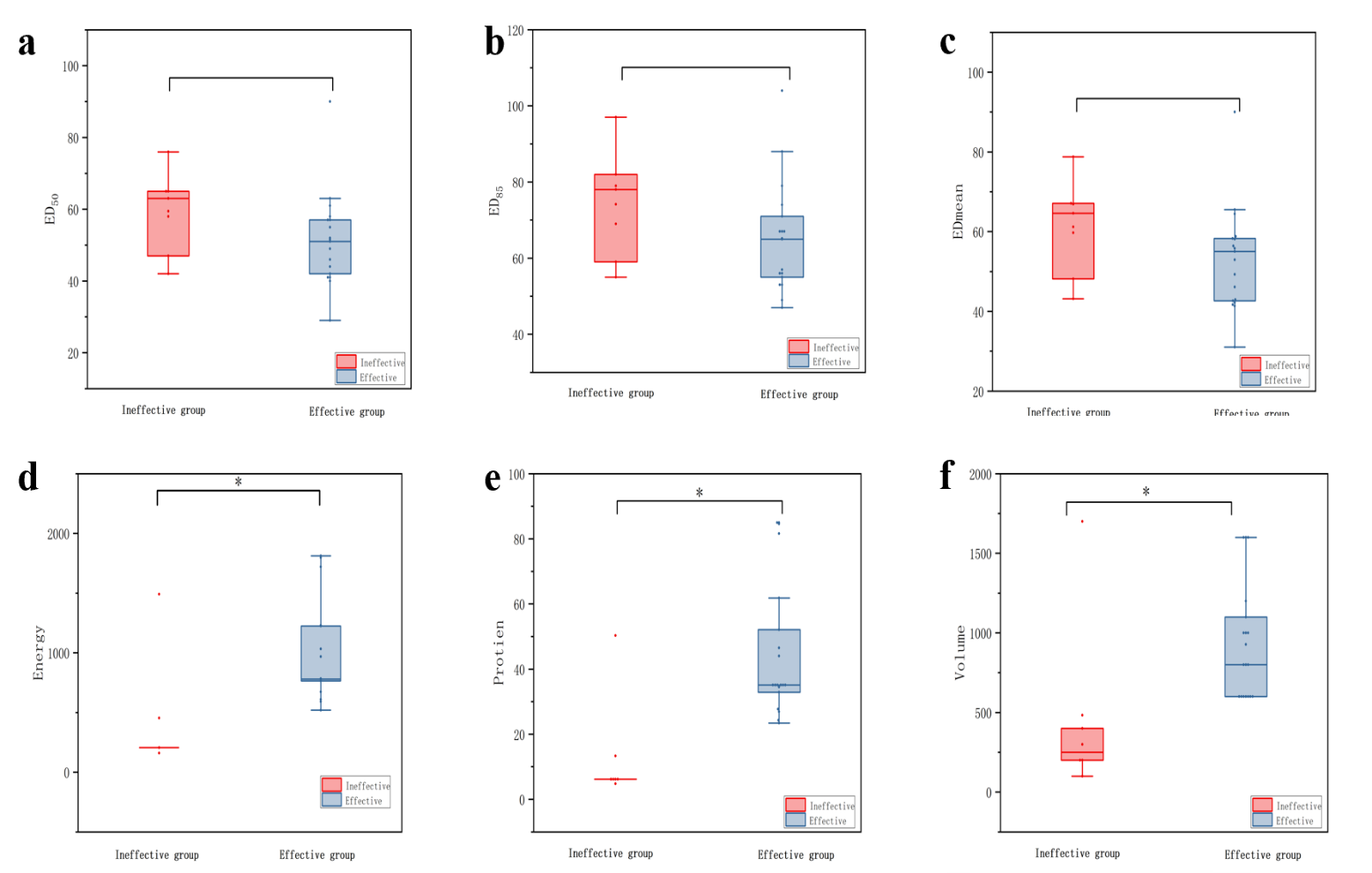


.

a, b and c representing the ED_50_, ED_85_, ED_mean_ between Effective group and Ineffective group using the domperidone, metoclopramide and mosapride agents respectively.

d, e and f representing the energy, protein and volume between Effective group and Ineffective group using the domperidone, metoclopramide and mosapride agents respectively.

**
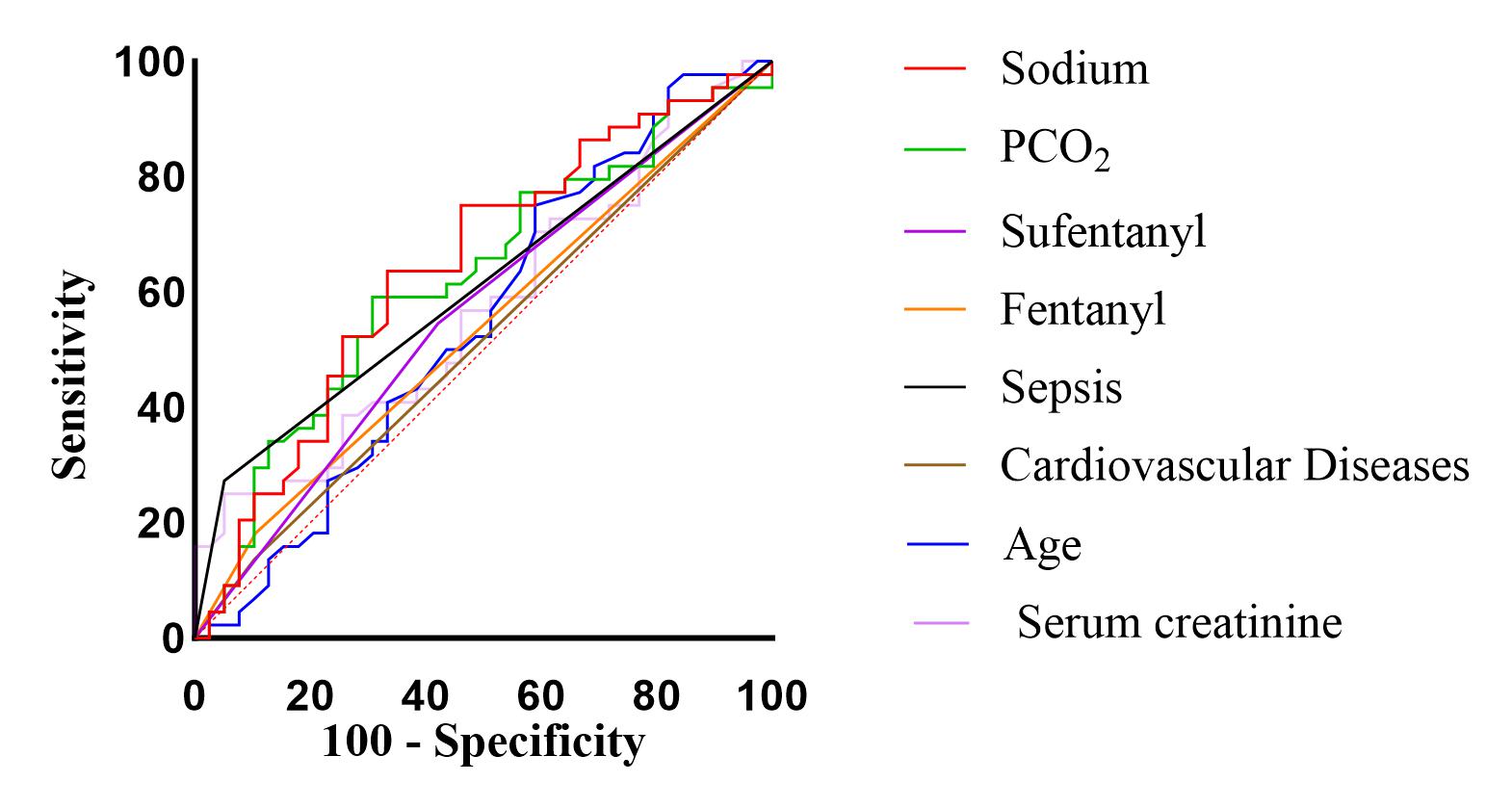
Supplementary Fig.11.**Receiver operator curve (ROC) for different risk factors (Age, Sepsis, Cardiovascular Diseases, Sufentanyl and Fentanyl, PCO2, and Sodium)

The cut off value of sodium was 136 and receiver operator curve for sodium was 0.644 (95%CI:52.43% to 76.42%, P=0.0239), sensitivity of 66.67% (95%CI:49.80% - 80.90%) and specificity of 63.64 % (47.80%- 77.60%); for PCO2 was 37.5 and ROC was 0.624 (95%CI 51.00 to 72.80, P=0.047), sensitivity of 69.23% (95%CI 52.4% - 83.0%) and specificity of 59.09% (95%CI 43.20% - 73.70%); for sepsis, was 0.611(95% CI 48.98% to 73.16%, P= 0.039), sensitivity of 94.87% (95%CI: 62.70 – 87.5%) and specificity of 27.27 (95% CI: 15.0% - 42.8%); for cardiovascular diseases, was 0.517(95% CI 40.50 to 62.80%, P=0.638), sensitivity of 89.74% (95%CI 65.8 %- 77.1%) and specificity of 13.64% (95%CI: 15.2% - 27.4%);for Fentanyl, was 0.538(95% CI:42.50 to 64.90, P= 0.323, sensitivity of 89.47% (95%CI: 75.8 - 87.1%) and specificity of 18.18% (95%CI: 8.20% - 32.70%); for Sufentanyl, was 0.562 (95%CI: 44.8 0% to 67.20%, P=0.2630, sensitivity of 57.89% (95%CI:40.80% - 73.70 %) and specificity of 54.55% (95%CI 38.80- 69.60%).

**Supplementary Table 1.** The logistic regression analysis to test the efficacy of prokinetic agents with ED_85_

| Variable | Unadjusted OR (95%CI) | Unadjusted P value | Model 1 | | Model 2 | | Model 3 | |
| --- | --- | --- | --- | --- | --- | --- | --- | --- |
|  |  |  | Odds Ratio (95%CI) | P value | Odds Ratio (95%CI) | P value | Odds Ratio (95%CI) | P value |
| ED85 | 1.043 (1.030-1.056） | <0.001 | 1.041 (1.028-1.055) | <0.001 | 1.043 (1.029-1.057) | <0.001 | 1.042 (1.027-1.057) | <0.001 |
| Age | 1.017 (1.006-1.028) | 0.002 | 0.994 (0.980-1.007) | 0.917 | 0.997 (0.983-1.010) | 0.620 | 0.993 (0.977-1.008) | 0.353 |
| Sepsis | 4.579 (2.692-7.790) | <0.001 | 5.210 (2.859-9.485) | <0.001 | 5.738 (3.102-10.617) | <0.001 | 4.865 (2.256-9.637) | <0.001 |
| Cardiovascular Diseases | 2.406 (1.340-4.317) | 0.003 | 3.423 (1.835-6.384) | <0.001 | 4.201 (2.231,7.913) | <0.001 | 3.433 (1.709-6.898) | <0.001 |
| Sufentanyl | 1.445 (1.102-2.063) | 0.043 |  |  | 4.007 (2.203-7.288) | 0.001 | 2.351 (1.510-3.658) | <0.001 |
| Fentanyl | 2.509 (1.456-4.324) | 0.001 |  |  | 2.054(1.350-3.126) | <0.001 | 3.886 (2.059-7.335) | <0.001 |
| Serum creatinine | 1.004 (1.002-1.006) | <0.001 |  |  |  |  | 1.002 (0.999-1.004) | 0.137 |
| PCO2 | 1.063 (1.040-1.087) | <0.001 |  |  |  |  | 1.043 (1.018-1.068) | 0.001 |
| Sodium | 1.076 (1.043-1.110) | <0.001 |  |  |  |  | 1.070 (1.032-1.111) | <0.001 |

Model 1 was adjusted for age, Sepsis, Cardiovascular Diseases. Model 2 was adjusted for age, Sepsis, Cardiovascular Diseases, Sufentanyl and Fentanyl. Model 3 was adjusted for age, Sepsis, Cardiovascular Diseases, Sufentanyl and Fentanyl, Serum creatinine, PCO2 and Sodium.

There is collinearity among ED_50_, ED_85_ and EDmean and cannot be included in the same model. In Model 1, 2, and 3, the results for these variables were generated by only entering the ED_50_ into the multivariable-adjusted model. Among them, Sepsis and Cardiovascular Diseases were the reason for admission to ICU; Sufentanyl and Fentanyl were the frequency of patients during the observational period; the level of Serum creatinine, PCO2 and Sodium were obtained at ICU admission.

**Supplementary Table 2.** The logistic regression analysis to test the efficacy of prokinetic agents with ED_mean_

| Variable | Unadjusted OR (95%CI) | Unadjusted P value | Model 1 | | Model 2 | | Model 3 | |
| --- | --- | --- | --- | --- | --- | --- | --- | --- |
|  |  |  | Odds Ratio (95%CI) | P value | Odds Ratio (95%CI) | P value | Odds Ratio (95%CI) | P value |
| EDmean | 1.050 (1.036-1.065) | <0.001 | 1.048 (1.033-1.064) | <0.001 | 1.044 (1.028-1.060) | <0.001 | 1.046 (1.029-1.063) | <0.001 |
| Age | 1.017 (1.006-1.028) | 0.002 | 0.993 (0.980-1.006) | 0.308 | 0.992 (0.978-1.006) | 0.268 | 0.991 (0.976-1.006) | 0.250 |
| Sepsis | 4.579 (2.692-7.790) | <0.001 | 5.249 (2.877-9.576) | <0.001 | 9.758 (4.116-14.621) | <0.001 | 6.579 (3.167-13.669) | <0.001 |
| Cardiovascular Diseases | 2.406 (1.340-4.317) | 0.003 | 3.342 (1.835-6.416) | 0.003 | 4.443(2.331-8.67) | <0.001 | 4.288 (2.096-8.771) | <0.001 |
| Sufentanyl | 1.445 (1.102-2.063) | 0.043 |  |  | 2.046(1.342-3.119) | 0.001 | 2.153(1.368-3.391) | 0.001 |
| Fentanyl | 2.509 (1.456-4.324) | 0.001 |  |  | 2.011 (1.265-3.713) | <0.001 | 3.205(1.651-6.222) | 0.001 |
| Serum creatinine | 1.004 (1.002-1.006) | <0.001 |  |  |  |  | 1.002 (1.000-1.006) | 0.038 |
| PCO2 | 1.063 (1.040-1.087) | <0.001 |  |  |  |  | 1.016 (0.991-1.042) | 0.210 |
| Sodium | 1.076 (1.043-1.110) | <0.001 |  |  |  |  | 1.104 (1.061-1.150) | <0.001 |

Model 1 was adjusted for age, Sepsis, Cardiovascular Diseases. Model 2 was adjusted for age, Sepsis, Cardiovascular Diseases, Sufentanyl and Fentanyl. Model 3 was adjusted for age, Sepsis, Cardiovascular Diseases, Sufentanyl and Fentanyl, Serum creatinine, PCO2 and Sodium.

There is collinearity among ED_50_, ED_85_ and EDmean and cannot be included in the same model. In Model 1, 2, and 3, the results for these variables were generated by only entering the ED_50_ into the multivariable-adjusted model. Among them, Sepsis and Cardiovascular Diseases were the reason for admission to ICU; Sufentanyl and Fentanyl were the frequency of patients during the observational period; the level of Serum creatinine, PCO2 and Sodium were obtained at ICU admission.

**Supplementary Table 3.** Baseline and characteristics of patients in prokinetic agent of metoclopramide

| **Characteristic** | **Total**  **(n=10)** | **Effective Group**  **(n=2)** | **Ineffective Group**  **(n=8)** | | |
| --- | --- | --- | --- | --- | --- |
| Age (years), mean ± SD | 53.10 ± 18.75 | 65.00 ± 15.56 | 50.13 ± 19.16 | | |
| Male Sex, No. (%) | 9 (90) | 2 (100.00) | 7 (87.50) | | |
| BMI, mean ± SD | 25.14 ± 4.28 | 23.69 ± 1.55 | 25.50 ± 4.74 | | |
| APHACHE II score, mean ± SD | 18.20 ± 9.83 | 26.50 ± 3.53 | 16.13 ± 9.89 | | |
| SOFA score, mean ± SD | 11.00 ± 3.62 | 10.50 ± 3.53 | 11.13 ± 3.87 | | |
| Ultrasonic parameters during the observational period, mean ± SD | | | |  |  |
| ED_50_ | 56.91 ± 13.25 | 47.00 ± 8.55 | 59.25 ± 13.14 | | |
| ED_85_ | 73.19 ± 14.42 | 61.38 ± 11.32 | 75.98 ±13.71 | | |
| ED_mean_ | 59.03 ± 12.96 | 50.15 ± 10.32 | 61.13 ± 12.70 | | |
| Nutrition during the observational period, mean ± SD | | | | | |
| Protein | 27.38 ± 23.40 | 60.01 ± 19.75 | 19.66 ± 16.58 | | |
| Energy | 591.49 ± 469.18 | 1210.99± 405.16 | 447.19 ± 354.55 | | |
| Volume | 612.50 ± 409.35 | 1076.32 ± 332.04 | 502.73 ± 345.261 | | |
| Length of ICU stay, mean ± SD, day | 20.20 ± 16.21 | 14.50 ± 7.78 | 21.63 ± 17.83 | | |
| Length of hospital stay, mean ± SD, day | 27.28 ± 24.29 | 17.16 ± 13.91 | 29.81 ± 26.35 | | |
| 28d mortality (%) | 3 (30) | 0 (0) | 3 (37.50) | | |
| In-hospital mortality (%) | 4 (40) | 1 (50) | 3 (37.50) | | |
| The days of prokinetic agents | 6.30 ± 5.45 | 6.50 ± 6.36 | 6.25 ± 5.70 | | |
| The rate of placing nasointestinal tube after using the agents, No. (%) | 4 (40) | 0 (0) | 4 (50.00) | | |

**Supplementary Table 4.** Baseline and characteristics of patients in prokinetic agent of domperidone

| **Characteristic** | **Total**  **(n=7)** | **Effective Group**  **(n=2)** | | **Ineffective Group**  **(n=5)** | |
| --- | --- | --- | --- | --- | --- |
| Age (years), mean ± SD | 57.57 ± 11.69 | 49.00 ± 12.72 | | 61.00 ± 10.63 | |
| Male Sex, No. (%) | 6 (85.71) | 2(100) | | 4 (80) | |
| BMI, mean ± SD | 27.30 ± 2.65 | 27.40 ± 5.28 | | 27.26 ± 1.87 | |
| APHACHE II score, mean ± SD | 17.43 ± 5.91 | 16.50 ± 2.12 | | 17.80 ± 7.12 | |
| SOFA score, mean ± SD | 12.00 ± 4.28 | 15.00 ± 4.24 | | 10.80 ± 4.08 | |
| Ultrasonic parameters during the observational period, mean ± SD | | |  | |  |
| ED_50_ | 58.85 ± 17.59 | 55.57 ± 18.27 | | 60.62 ± 17.31 | |
| ED_85_ | 75.35 ± 19.88 | 71.86 ± 22.28 | | 77.23 ± 18.66 | |
| ED_mean_ | 60.52 ± 17.53 | 56.91 ± 18.18 | | 62.46 ± 17.21 | |
| Protein | 23.94 ± 16.99 | 36.80 ± 12.91 | | 17.02 ± 14.86 | |
| Nutrition during the observational period, mean ± SD | | | | | |
| Energy | 583.66 ± 434.44 | 1026.26 ± 288.09 | | 325.33 ± 287.22 | |
| Volume | 593.75 ± 435.92 | 992.86 ± 377.16 | | 378.85 ± 291.95 | |
| Length of ICU stay, mean ± SD, day | 21.71 ± 12.76 | 29.50 ±19.09 | | 18.60 ± 10.52 | |
| Length of hospital stay, mean ± SD, day | 37.46 ± 16.34 | 40.91 ± 3.33 | | 35.74 ± 20.71 | |
| 28d mortality (%) | 2 (28.57) | 0 (0) | | 2 (40) | |
| In-hospital mortality (%) | 3 (42.86) | 1 (50) | | 2 (40) | |
| The days of prokinetic agents | 11.00 ± 6.16 | 10.00 | | 11.25 ± 7.09 | |
| The rate of placing nasointestinal tube after using the agents, No. (%) | 1 (14.29) | 0 (0) | | 1 (20) | |

**Supplementary Table 5.** Baseline and characteristics of patients in prokinetic agent of mosapride

| **Characteristic** | **Total**  **(n=35)** | **Effective Group**  **(n=23)** | **Ineffective Group**  **(n=12)** | | |
| --- | --- | --- | --- | --- | --- |
| Age (years), mean ± SD | 57.34 ± 16.61 | 56.39 ± 19.01 | 59.17 ± 19.01 | | |
| Male Sex, No. (%) | 25 (71.42) | 17(73.91) | 8 (66.67) | | |
| BMI, mean ± SD | 22.10 ± 3.76 | 21.95 ± 3.88 | 22.39 ± 3.66 | | |
| APHACHE II score, mean ± SD | 15.51 ± 8.42 | 15.13 ± 7.87 | 16.25 ± 8.69 | | |
| SOFA score, mean ± SD | 9.51 ± 3.99 | 9.13 ± 3.99 | 10.25 ± 4.07 | | |
| Ultrasonic parameters during the observational period, mean ± SD | | | |  |  |
| ED_50_ | 51.83 ± 13.83 | 48.23 ± 12.34 | 57.76 ± 14.18 | | |
| ED_85_ | 67.81 ± 15.91 | 63.95 ± 14.63 | 74.19 ± 15.95 | | |
| ED_mean_ | 53.81 ± 14.15 | 50.34 ± 12.92 | 59.55 ± 14.29 | | |
| Nutrition during the observational period, mean ± SD | | | | | |
| Protein | 31.33 ± 20.24 | 38.47 ± 16.86 | 19.55 ± 19.91 | | |
| Energy | 744.18 ± 465.97 | 938.56 ± 389.91 | 424.14 ± 400.29 | | |
| Volume | 758.69 ± 446.21 | 955.10 ± 353.10 | 434.27 ± 392.37 | | |
| Length of ICU stay, mean ± SD, day | 16.07 ± 7.89 | 17.56 ± 8.70 | 13.33 ± 5.48 | | |
| Length of hospital stay, mean ± SD, day | 29.87 ± 19.96 | 33.55 ± 20.00 | 23.12 ± 15.35 | | |
| 28d mortality (%) | 6 (17.14) | 3 (13.04) | 3 (25.00) | | |
| In-hospital mortality (%) | 11 (31.43) | 8 (34.78) | 3 (25.00) | | |
| The days of prokinetic agents | 7.94 ± 5.27 | 8.52 ± 5.88 | 6.70 ± 3.65 | | |
| The rate of placing nasointestinal tube after using the agents, No. (%) | 4 (11.43) | 0 (0) | 4 (33.33) | | |

**Supplementary Table 6.** Baseline and characteristics of patients in prokinetic agents of metoclopramide and mosapride

| **Characteristic** | **Total**  **(n=16)** | **Effective Group**  **(n=5)** | **Ineffective Group**  **(n=11)** | | |
| --- | --- | --- | --- | --- | --- |
| Age (years), mean ± SD | 51.00 ± 16.44 | 39.20 ± 12.31 | 56.36 ± 16.60 | | |
| Male Sex, No. (%) | 8 (50.00) | 1 (20) | 7 (63.63) | | |
| BMI, mean ± SD | 23.24± 5.47 | 23.78 ± 7.94 | 22.99 ± 4.42 | | |
| APHACHE II score, mean ± SD | 17.56 ± 8.27 | 19.60 ± 8.08 | 16.60 ± 8.58 | | |
| SOFA score, mean ± SD | 8.56 ± 3.68 | 9.60 ± 3.36 | 8.09 ± 3.88 | | |
| Ultrasonic parameters during the observational period, mean ± SD | | | |  |  |
| ED_50_ | 53.06 ± 12.27 | 49.05 ± 12.50 | 55.73 ± 11.47 | | |
| ED_85_ | 69.30 ± 14.89 | 66.35 ± 12.79 | 73.46 ± 14.82 | | |
| ED_mean_ | 55.08 ± 12.70 | 50.60 ± 12.79 | 58.05 ± 11.83 | | |
| Nutrition during the observational period, mean ± SD | | | | | |
| Protein | 30.26 ± 23.09 | 42.45 ± 19.62 | 21.87 ± 23.41 | | |
| Energy | 640.35 ± 501.71 | 892.42 ± 454.89 | 472.30 ± 462.22 | | |
| Volume | 627.14 ± 457.79 | 828.57 ± 376.30 | 492.86 ± 460.527 | | |
| Length of ICU stay, mean ± SD, day | 22.56 ± 10.18 | 26.80 ± 15.71 | 20.64 ± 6.62 | | |
| Length of hospital stay, mean ± SD, day | 37.96 ± 24.12 | 31.59 ± 16.96 | 40.86 ± 26.91 | | |
| 28d mortality (%) | 3 (18.75) | 1 (20.00) | 2 (18.18) | | |
| In-hospital mortality (%) | 4 (25.00) | 1 (20.00)) | 3 (27.27) | | |
| The days of prokinetic agents | 10.75 ± 5.46 | 12.67 ± 5.68 | 10.11 ± 5.57 | | |
| The rate of placing nasointestinal tube after using the agents, No. (%) | 4 (25.00) | 0 (0) | 4 (41.67) | | |

**Supplementary Table 7.** Baseline and characteristics of patients in prokinetic agents of domperidone and mosapride

| **Characteristic** | **Total**  **(n=11)** | **Effective Group**  **(n=3)** | **Ineffective Group**  **(n=8)** | | | |
| --- | --- | --- | --- | --- | --- | --- |
| Age (years), mean ± SD | 60.25 ± 20.50 | 53.50 ± 14.43 | 64.00 ± 11.72 | | |  |
| Male Sex, No. (%) | 8 (72.72) | 2 (50.00) | 6 (85.71) | | |  |
| BMI, mean ± SD | 26.28 ± 3.34 | 23.71 ± 1.82 | 22.44 ± 3.52 | | |  |
| APHACHE II score, mean ± SD | 20.25 ± 4.19 | 17.75 ± 4.57 | 22.14 ± 11.87 | | |  |
| SOFA score, mean ± SD | 8.50 ± 3.11 | 9.75 ± 0.96 | 10.00 ± 2.52 | | |  |
| Ultrasonic parameters during the observational period, mean ± SD | | | |  |  |  |
| ED_50_ | 54.67 ± 14.30 | 47.14 ± 10.53 | 58.64 ± 14.51 | | |  |
| ED_85_ | 70.96 ± 15.38 | 63.79 ± 12.46 | 74.75 ± 15.52 | | |  |
| ED_mean_ | 57.11 ± 13.63 | 49.12 ± 10.46 | 61.33 ± 13.38 | | |  |
| Nutrition during the observational period, mean ± SD | | | | | |  |
| Protein | 29.02 ± 23.53 | 44.33 ± 21.01 | 20.93 ± 20.72 | | |  |
| Energy | 645.52 ± 486.48 | 1001.70 ± 418.26 | 457.35 ± 410.92 | | |  |
| Volume | 624.07 ± 428.45 | 925.09 ± 348.67 | 456.09 ± 380.48 | | |  |
| Length of ICU stay, mean ± SD, day | 26.00 ± 15.74 | 39.75 ± 20.18 | 19.43 ± 8.77 | | |  |
| Length of hospital stay, mean ± SD, day | 55.41± 58.99 | 54.49 ± 31.31 | 35.86 ± 14.51 | | |  |
| 28d mortality (%) | 3 (27.27) | 0 (0) | 3 (27.27) | | |  |
| In-hospital mortality (%) | 4 (33.36) | 1 (25.00) | 3 (27.27) | | |  |
| The days of prokinetic agents | 17.25 ± 10.21 | 15.25 ± 8.02 | 8.17 ± 5.08 | | |  |
| The rate of placing nasointestinal tube after using the agents, No. (%) | 5 (38.46) | 0 (0) | 5 (71.43) | | |  |

**Supplementary Table 8.** Baseline and characteristics of patients in prokinetic agents of domperidone, metoclopramide and mosapride

| **Characteristic** | **Total**  **(n=4)** | **Effective Group**  **(n=3)** | **No-Effective Group**  **(n=1)** | |
| --- | --- | --- | --- | --- |
| Age (years), mean ± SD | 60.25 ± 20.50 | 66.33 ± 20.21 | 42.00 | |
| Male Sex, No. (%) | 2 (50.00) | 1 (33.33) | 1 (100.00) | |
| BMI, mean ± SD | 26.28 ± 3.34 | 27.05 ± 3.64 | 24.00 | |
| APHACHE II score, mean ± SD | 20.25 ± 4.19 | 20.33 ± 5.13 | 20.00 | |
| SOFA score, mean ± SD | 8.50 ± 3.11 | 8.00 ± 3.61 | 10.00 | |
| Ultrasonic parameters during the observational period, mean ± SD | | |  |  |
| ED_50_ | 53.83 ± 13.16 | 51.53 ± 13.38 | 59.43 ± 11.62 | |
| ED_85_ | 67.79 ± 15.05 | 65.18 ± 14.93 | 74.14 ± 14.40 | |
| ED_mean_ | 55.33 ± 13.39 | 52.92 ± 13.47 | 61.19 ± 12.14 | |
| Nutrition during the observational period, mean ± SD | | | | |
| Protein | 36.33 ± 22.84 | 44.00 ± 20.56 | 13.32 ± 18.13 | |
| Energy | 829.67 ± 499.67 | 968.75 ± 416.57 | 412.42 ± 529.05 | |
| Volume | 816.67 ± 465.94 | 927.78 ± 365.91 | 483.33 ± 604.70 | |
| Length of ICU stay, mean ± SD, day | 26.00 ± 15.75 | 18.67 ± 7.02 | 48.00 | |
| Length of hospital stay, mean ± SD, day | 26.67 ± 16.77 | 26.67 ± 16.77 | 141.00 | |
| 28d mortality (%) | 0 (0) | 0 (0) | 0 (0) | |
| In-hospital mortality (%) | 0 (0) | 0 (0) | 0 (0) | |
| The days of prokinetic agents | 17.25 ± 10.21 | 12.6 ± 5.51 | 31.00 | |
| The rate of placing nasointestinal tube after using the agents, No. (%) | 1 (25.00) | 0 (0) | 1 (100.00) | |
